# Dietary inadequacy in Tanzania is linked to the rising cost of nutritious foods and consumption of food-away-from-home

**DOI:** 10.1016/j.gfs.2023.100679

**Published:** 2023-06

**Authors:** Liz Ignowski, Ben Belton, Nhuong Tran, Hannah Ameye

**Affiliations:** a WorldFish, Phnom Penh, Cambodia; b Michigan State University, East Lansing, MI, USA; c International Food Policy Research Institute, Dhaka, Bangladesh; d WorldFish, Penang, Malaysia; e LICOS Centre for Institutions and Economic Performance, KU Leuven, Belgium

**Keywords:** Tanzania, Cost of the diet, Food prices, Micronutrient adequacy, Nutrition

## Abstract

This study contributes to the growing literature on dietary quality and accessibility in the Global South. We analyze the nutrition implications of changing dietary patterns between 2008 and 2019 in one of Africa's largest and fastest growing economies, Tanzania, and compare patterns at national and sub-national scales. We find that: (1) Rising incomes have not been associated with marked increases in the diversity of food consumed at home; (2) Consumption of food away from home has increased dramatically; (3) Most food consumed in Tanzanian homes is purchased instead of self-produced; (4) There have not been clear improvements in the adequacy of micronutrient consumption obtained from food eaten at home; (5) The most affordable sources of key micronutrients, including nutrient dense foods such as dried fish, have become more expensive. Our findings indicate that in Tanzania the amount and diversity of nutritious foods eaten at home have not improved with rising incomes, but consumption of energy-dense processed foods eaten away from home has increased rapidly, likely reflecting differences in convenience and relative prices. To improve Tanzanian diets in coming years, coordinated nutrition-sensitive policy actions will be required on both the supply- and demand-sides.

## Highlights

• We analyze nutrition implications of changing diets in Tanzania 2008–2019.
• Rising incomes did not increase the diversity of food consumed at home.
• Consumption of unhealthy food away from home increased dramatically.
• Micronutrient adequacy did not improve.
• The most affordable sources of micronutrients became more expensive.

## 1 Introduction

Malnutrition remains a key global development challenge. Most countries are not on track to meet the ‘Zero Hunger’ Sustainable Development Goal (SGD) by 2030 (von Grebmer et al., 2020). There are an estimated 1.5 billion micronutrient deficient people globally (World Health Organization, 2018) and this number has grown since 2020 due to COVID-19 raising the number of undernourished people in the world by over 100 million from 2019 to 2020 (UNICEF, 2021).

Bennett's Law (Bennett, 1941) is a widely observed pattern whereby the proportion of energy, expressed as kilocalories (kcal), derived from starchy staples falls as incomes rise (Timmer et al., 1983). Evidence of this trend has been found across globe, including in Africa (Desiere et al., 2018; Tschirley et al., 2015). As incomes increase, consumers tend to diversify their food consumption to include a higher share of non-staples, causing the share of food expenditure allocated to more expensive nutritious food such as fruits, vegetables, meat, dairy, and fish to rise disproportionately (Reardon et al., 2014).

In much of the Global South, including some African countries, the growing ‘downstream pull’ of demand for a diversified diet has given rise to the rapid transformation in the upstream and midstream of agri-food supply chains supplying cities, via the proliferation of increasingly specialized commercially oriented farms, and enterprises providing these farms with goods and services (Reardon, 2015; Reardon et al., 2012).

Processes of structural transformation, urbanization, and deepening market integration that drive rising incomes and consumer demand also create conditions for the proliferation of large- and small-scale processing and retailing of convenience foods (Reardon et al., 2021; Sauer et al., 2021). These foods, which are often eaten away from home, tend to contain high levels of sugars, fats, and salt, but few micronutrients (Popkin, 1998; Reardon et al., 2021; Sauer et al., 2021). As a result, obesity has increased with economic growth in many low- and middle-income countries (LMIC) as consumption of low nutritional value processed foods has risen in combination with reductions in physical activity, while undernutrition remains persistent (Ameye and Swinnen, 2019). These conditions give rise to the ‘triple burden of malnutrition’, whereby undernutrition (stunting, wasting and underweight), micronutrient deficiencies, and over nutrition (overweight and obesity) coexist in a single locale, and sometimes within the same household (Sunuwar et al., 2020).

Attempts to account for this paradox have drawn attention to the ‘cost of the diet’ – i.e., the monetary cost of acquiring a nutritionally adequate diet, accounting for location-specific food availability, cultural preferences, and prevailing prices (Bai et al., 2021; Mahrt et al., 2019; Masters et al., 2018; Schneider et al., 2021; Webb et al., 2020). This growing body of work suggests that diets that provide adequate nutrition are unaffordable to a large share of the population in many LMIC. A meta-analysis of relative caloric prices for different categories of food across 176 countries found that in low-income countries, healthy foods were generally expensive (Headey and Alderman, 2019). For example, in Kenya, Tanzania, and Uganda, over 90% of people cannot afford the healthy diet as defined by EAT-Lancet (Headey et al., 2021), while studies from Ethiopia, Malawi, and Tanzania have found that seasonal food price fluctuations make nutritional adequacy difficult to achieve, even with substitution among nutrient sources (Bai et al., 2020). In addition to seasonal price variations, these authors observed large regional variations in food prices.

A related body of literature explores the temporal evolution of food prices. This body of work suggests that real prices of many nutritious foods are becoming more expensive over time, relative to less healthy processed foods and/or in absolute terms - e.g., see evidence from Ethiopia (Ameye et al., 2021), Brazil, China, Mexico and Korea (Wiggins et al., 2015), and South Asia (Dizon and Herforth, 2018). Recent work on consumer preferences in Tanzania found that prices of staple foods such as maize, which is highly consumed there, also influence diet quality (McCullough et al., 2022).

Drawing together these strands, we study dietary changes in Tanzania, which is one of sub-Saharan Africa's fastest growing economies, with annual GDP growth averaging 6% since 2000 (World Bank, 2022). However, Tanzania also has one of the highest undernourishment rates globally, ranking 89th out of 107 countries (von Grebmer et al., 2020). Approximately one-quarter of the population lives below the national poverty line, about half the population is moderately food-insecure, and the country continues to face food shortages (FAO, 2013; Mkonda and He, 2018; World Bank, 2022). Further, recent work by Beal et al. (2022) found that of 13 sub-Saharan African countries surveyed, Tanzania had the highest risk of micronutrient deficiency for women. At the same time, Tanzanian women experienced a 20% increase in obesity levels between 2012 and 2016 alone, going from a prevalence of 8.9%–10.7% respectively (WHO, 2016).

We utilize data from consumption modules from five rounds of a nationally representative household living standards measurement survey, the Tanzania National Panel Surveys (NPS), implemented between 2008 and 2019, to study changes over time in: (1) Dietary composition (frequency, quantity, and source of foods consumed, and share of total micronutrients originating from different food groups); (2) Adequacy of consumption of energy, protein, calcium, zinc, iron, vitamin A and vitamin B12, relative to estimated average requirements; (3) The evolving cost of foods and the micronutrients derived from them. In all cases, we compare patterns at the national and sub-national level, including rural and urban areas, and six agro-ecological zones with distinct socio-economic characteristics. This approach allows for interpretation of consumption patterns in relation to spatial variables, including geography, agro-ecology, and cultural preference, and offers a more granular analysis than previous studies (e.g., Keding, 2016).

In addition to the NPS data, used to track the quantities and prices of foods consumed at home, we use two other data sources to study the nutritional implications of changing dietary patterns, namely: (1) The Tanzania Food Composition Tables, which provide information on the nutrient composition of foods (Lukmanji et al., 2008); (2) Daily food acquisition diaries from Tanzania, to fine-tune which foods are most commonly consumed within the aggregate food groups reported in NPS (Ameye et al., 2021). Combining this information, we estimate changes over time in the cost and quantities of food items consumed at home, the price of micronutrients obtained from those food items, and the adequacy of micronutrient consumption relative to recommended intakes. Furthermore, following the methodology set out by Moltedo et al. (2018), we impute the energy content of food eaten away from home, expressed as kcal.

We find the following: First, contrary to Bennett's Law, rising average real incomes have not been associated with marked increases in the diversity or quantity of non-staple foods consumed at home. Second, the share of expenditure on food away from home (FAFH) jumped 42% over the survey period, though with wide variations by zone. The survey data do not allow for estimation of the micronutrient composition of FAFH, but evidence from other studies in Tanzania indicates that FAFH is commonly ultra-processed, high in oils and fats, and low in micronutrients, thus generally unhealthy (Ameye, 2020; Cockx et al., 2018; Sauer et al., 2021). Third, throughout the survey period, most of the food consumed at home in Tanzania was purchased, including in rural areas. Fourth, there have not been clear improvements in the adequacy of micronutrient consumption from food eaten at home over the study period, despite small differences between zones. Fifth, the most affordable sources of micronutrients are becoming more expensive. Real prices increased across all food groups, with meat, fish, and fruit all increasing the most, by almost 50% each on average from 2008 to 2019. This trend appears to have dampened consumption and nutrition gains that might otherwise have been expected due to rising incomes.

Although incomes have risen in Tanzania, dietary improvements have not occurred as expected. Diet diversity in the home has changed little over time, resulting in minimal changes in micronutrient consumption. This behavior is likely linked to our finding that some of the most affordable sources of micronutrients are rapidly becoming more expensive. Instead of consuming healthier and more diverse diets at home, Tanzanians are consuming increasing quantities of foods away from home, which may not be the healthiest options.

Tanzania's food system is thus reaching a critical juncture. Simultaneous actions and interventions are required on the supply side to increase the availability of healthy foods at affordable prices, and on the demand side, to increase preferences for and access to more diverse healthy foods, whether consumed in the home or outside it.

The rest of this paper is organized as follows: Section 2 presents our data and methodologies, Section 3 presents our findings, and Section 4 concludes with a discussion of policy implications.

## 2 Data and methodology

Our analysis combines multiple datasets and methodologies. Our main data set is publicly available household survey data from the nationally representative Tanzania National Panel Survey (NPS). We utilize five rounds of this survey: 2008, 2010, 2012, 2014, and 2019. The survey is comprised of four structured questionnaires (household, agriculture, livestock, and community), each consisting of several modules. The surveys collected data on topics including household demographic characteristics, income sources, assets, food and non-food expenditures, and food consumption.

The survey was a panel survey for rounds collected in 2008, 2010, and 2012. A new sample was selected in 2014 and 2019 but both included a sub-sample from the original rounds. While some households are included in multiple rounds, we treat each round as a nationally representative cross-sectional sample, after applying survey weights. Our total sample size is 16,639 households distributed over five rounds across an 11-year period: 2008: 3222; 2010: 3902; 2012: 4991; 2014: 3344; 2019: 1180.

Tanzania's geography is diverse, and the country is divided into six primary agro-ecological zones; the Coastal plains, Northern Highlands, Lake zone, Central arid plains, Southern Highlands, and the Southern zone (The United Republic of Tanzania, 2017). The Coastal and Lake zones have the highest population densities as they are the most urbanized, with the cities of Dar es Salaam and Mwanza, respectively. Approximately 40% of the population live in the Coastal zone and 30% live in the Lake zone. Table A1 in the annex presents the sample size by urban, rural, and geographic zone over survey rounds. Table A2 presents the share of urban households by geographic zone over survey rounds. Consumption data were collected in every month in most geographic zones and all but one survey round (2019). Given the survey design and large sample size, we are confident that the timing of data collection averages out the effects of seasonality on consumption.

Our main variables of interest from the NPS come from the food consumption module. The survey collected food consumption data using seven-day recall, validated independently by Ameye et al. (2021) as being a relatively reliable measure. The data include information on whether the food was obtained via purchase, own production, or gifted. The value of food not purchased was imputed using the median price for the region (there are 26 regions within the country). These prices were calculated from other respondents in the same region who had purchased these items.

The quantity of food items consumed is expressed in kilograms. For items reported in non-standard units, we used conversion factors where applicable (e.g., for eggs and chickens). When there were no potential conversions, we applied the median consumption amount for the zone (e.g., for bread and buns which were reported in ‘pieces’). Due to data being reported at the household level, we are not able to study intra-household consumption patterns. We control for the demographic composition of each household by calculating the number of adult equivalents (AE) per household, using the adult-equivalence scale, which accounts for age and gender of all household members, as reported by the National Bureau of Statistics (NBoS), 2011. Use of AEs is common practice in the literature (Ameye et al., 2021; Sauer et al., 2021; Van den Broeck et al., 2021) and allows for more accurate measures of indicators such as average household consumption that could be influenced by household demographics. For example, males aged 15–59 years of age equal one adult equivalent while females of the same age equal 0.88 adult equivalents, as women tend to consume less than men. Children, both male and female, have the same equivalency factors until the age of 10.

Food consumption at home in the NPS is reported for 59 categories of food, some of which combine several similar items (e.g., “pulses”, comprised of “peas, beans, lentils and other pulses”). Consumption data were reported by one household member for the entire household. In the absence of further information on the foods consumed, it would have been necessary to assign equal weights to each food item in aggregate food groups. However, we improved upon this data by utilizing more detailed consumption data from Ameye et al. (2021). These authors collected enumerator-assisted daily household food acquisition diary entries disaggregated into 99 food items, using Swahili food descriptions from 500 households over a 14-day period. The study was statistically representative of urban and rural areas in Tanzania and covered half of the geographical zones in our analysis.

This additional information allowed us to weight individual foods within the 59 aggregate groups more accurately than would have been possible using NPS data alone. For example, per the food diary dataset, the NPS category “beef” is comprised of 94% cooked beef, 3% beef liver and 3% beef tripe. Without this information, we would have had to equally weigh cooked beef, beef liver and beef tripe which would result in a very different nutritional composition, because beef liver contains much more Vitamin A than beef meat or tripe. Assuming equal weights for all three items would severely overestimate Vitamin A intake and underestimate Vitamin A deficiencies. A table of the food items and their corresponding weights is presented in Table A3 in the Annex.

After calculating the average daily food consumption per AE in our sample households, we estimated micronutrient consumption, using data from the Tanzania Food Composition Tables, which contain records of 47 nutrients in over 400 Tanzanian foods (Lukmanji et al., 2008). The quantity of nutrients provided by each food item listed in NPS was estimated by multiplying the weight of the food item by its nutrient concentration, as reported in the food composition tables.

We focused on energy (kcal) and protein, and the micronutrients calcium, iron, zinc, vitamin A, and vitamin B12. Energy is a common starting point for nutritional analysis and protein is the macronutrient most under-consumed in Sub-Saharan Africa (Ameye, 2020). We selected these micronutrients as they are some of the most common deficiencies (particularly in Tanzania) and those with data widely available (Abdulai and Aubert, 2004; Finaret and Masters, 2019; Van den Broeck et al., 2021). In particular, calcium is important for bone growth and cellular function, iron is the most common micronutrient deficiency and crucial for cellular respiration and cognitive function, zinc is essential for cellular metabolism, vitamin A is important for multiple functions in the body, and vitamin B12 is necessary for energy production and the nervous system (Ahern et al., 2021). Lastly, all are important for human growth, physical and mental development, and overall health (Bhutta et al., 2008; Black et al., 2008).

Given that the recommended intakes of each nutrient are different, we present the adequate consumption of nutrients normalized to the Estimated Average Requirement (EAR). The EAR is defined as the average daily nutrient intake that is estimated to meet the requirements of half of the healthy individuals of a given life stage and gender group (Institute of Medicine, 2006). This value is more appropriate for population level analysis than recommended intake, which is the average daily nutrient intake to meet the nutrient requirements of 98% of healthy individuals of a given life stage and gender group (Institute of Medicine, 2006). The values for EAR used to denote nutritional adequacy in this analysis are 2400 kcal, 50 g protein, 805 mg calcium, 10.4 mg iron, 10 mg zinc, 540 μg retinol activity equivalents (RAE) vitamin A, and 2 mg vitamin B12 (Allen et al., 2006). As energy requirements (kcal) cannot be calculated by the EAR method (Allen et al., 2006), we used values calculated by Waid et al. (2017) based on moderate physical activity level.

We also calculate the monetary value of food consumed. To adjust for changes in price linked to inflation and exchange rate fluctuations, the prices of all food items consumed in the household were converted to constant 2011 United States Dollars (USD) at purchasing-power parity (PPP) rates, similar to studies such as Hirvonen et al. (2020) and Van den Broeck et al. (2021). We calculated the amount spent on each food item, allowing us to create a price per nutrient consumed.

FAFH accounts for a growing share of food consumption throughout LMICs, including in Tanzania (Cockx et al., 2018). NPS collects data on household expenditures (collected at the individual level within the household) for only a few very broad categories of FAFH such as: full meals, snacks prepared on charcoal outside of the household, local brews, wine or other commercial spirits, sodas and non-alcoholic drinks, sweets, and tea, coffee, samosa, cake, and other snacks. These categories were consistent in all rounds of the survey. We utilized the total monetary value that each household spent on FAFH. These broad product categories make it impossible to estimate micronutrient consumption obtained from this component of the diet. We therefore use a method from Moltedo et al. (2018) to estimate energy consumed away from home.

The method of Moltedo et al. (2018) imputes kcals in FAFH using data on the median at-home cost-per-kcal. The medians are calculated by year at the regional level, urban-rural status, and income quintile levels for more precise estimates. We acknowledge that the calculation of energy consumed in FAFH using this method is an approximation, but contend that it allows for a more complete picture of the average diet within our sample population than would be possible otherwise. However, we felt that calculating protein and micronutrients consumption using this method would be excessively speculative, and did not attempt to do so.

Further, although we know which types of full meals are consumed from the diary data in Ameye et al. (2021), taking an average in order to suit the food groups defined by the NPS data would be too imprecise. For example, the food group “full meals” includes dishes like Ugali, a porridge dish, Maharage, a banana and kidney bean dish, and Wali, a coconut and rice dish. Each differs substantially in nutrient content. Also important for FAFH are snacks. This food group is even more diverse, including items such as donuts and cakes, fried plantain and beef skewers, and groundnut clusters and chickpea fritters. We therefore excluded these foods from our nutrient consumption calculations.

## 3 Results

### 3.1 General findings

Table 1 presents data from the five rounds of NPS from 2008 to 2019, disaggregated by national and rural/urban zones on: (1) Consumption expenditure at constant 2011 PPP USD per AE; (2) Share of consumption expenditure devoted to all food; (3) Share of consumption expenditure devoted to food eaten at home; (4) Share of consumption expenditure devoted to food eaten away from home; (5) Diet diversity scores (the average number of food groups consumed at home per household during the survey's seven-day recall period); (6) Degree of inequality in consumption expenditure across the whole sample (measured by the Gini coefficient). Consumption expenditure is the value of annual total household consumption (including the imputed value of own production consumed at home), calculated and made available as part of the dataset for each round of the survey by the NPS.

**Table 1:** National, rural, and urban household consumption expenditure, food as a share of consumption, dietary diversity, and consumption inequality by survey round, 2008–2019.

	Consumption expenditure USD (PPP)			Food as share of consumption expenditure (%)			Food eaten at home as share of consumption expenditure (%)		
	Tanzania	Rural	Urban	Tanzania	Rural	Urban	Tanzania	Rural	Urban
2008	1292	1015	2072***	77.8	81.5	67.4***	70.0	75.7	53.6***
2010	1397	1075	2120***	75.4	79.1	67.1***	64.7	71.5	49.4***
2012	1703	1253	2676***	75.1	79.3	66.0***	62.0	69.4	46.1***
2014	1713	1307	2518***	73.5	78.1	64.3***	62.1	69.4	47.9***
2019	1678	1346	2423***	72.7	76.8	63.6***	59.6	67.2	42.6***
	**FAFH as share of consumption expenditure (%)**			**Diet diversity score (Num. of food groups out of 12)**			**Gini coefficient**		
	**Tanzania**	**Rural**	**Urban**	**Tanzania**	**Rural**	**Urban**	**Tanzania**	**Rural**	**Urban**
2008	7.8	5.7	13.8***	8.0	7.7	8.8***	0.40	0.37	0.40***
2010	10.7	7.6	17.7***	8.2	8.0	8.6***	0.39	0.37	0.39***
2012	13.1	9.9	19.8***	8.0	7.8	8.5***	0.40	0.37	0.38
2014	11.3	8.7	16.5***	8.6	8.2	9.2***	0.39	0.37	0.37
2019	13.1	9.6	21.1***	8.0	7.9	8.2	0.39	0.39	0.37

Results are nationally representative with the use of survey weights. ***p < 0.01, **p < 0.05, and *p < 0.10. Urban statistics present the results of an adjusted Wald test which test the difference in rural/urban means year by year.

We find that average annual consumption expenditures increased over time for households in all zones, growing by 30% at the national level from 2008 to 2019, consistent with increasing average GDP per capita over this period. As expected, rural consumption was substantially lower than urban consumption ($1346 vs $2423/capita in 2019, at constant 2011 PPP prices). The share of household income allocated to food expenditures generally fell as incomes rose, a phenomenon referred to as Engle's Law. This tendency is observable in Tanzania during the survey period, but the effect is small at the national level and concentrated predominantly in rural areas, with substantial variations by geographical zone (Annex Table A4). The average share of the national household consumption budget allocated to food fell by 5.1 percentage points from 2008 to 2019, falling in both rural and urban areas, with the share spent on food always higher in rural areas (Table 1).

Breaking down household budget shares into food consumed at home and FAFH reveals a picture of rapid change. The share of food eaten at home in total household expenditure fell by just over 10 percentage points for Tanzania as a whole, from 70% to 59% over the survey period. The decline was slowest in rural areas (8 percentage points). In contrast, the share of FAFH in total household consumption climbed almost 6 percentage points for Tanzania as a whole, from 7.8% to 13.1% - a jump of 68% in relative terms. The share of household budget allocated to FAFH in rural areas increased from 5.7% to 9.6%; also a relative increase of 68%. FAFH expenditure in urban areas grew somewhat more slowly, by 53% in relative terms. Thus, although the share of national household budgets allocated to food decreased slightly, likely due to increases in income, this pattern masks extremely rapid change in the composition of food expenditure, with a rapid shift in consumption expenditures from foods eaten at home to FAFH.

As noted in the introduction, Bennett's Law predicts that as incomes rise, the share of energy derived from staple foods will fall as they are substituted for by more expensive, income elastic non-staple foods. Diets tend to diversify with rising income as a result. This tendency was not apparent for foods eaten at home in Tanzania during the survey period. Our diet diversity score uses the 12 food groups suggested in the widely used Household Dietary Diversity Score indicator developed by the Food and Agriculture Organization of the United Nations (FAO). This does not include FAFH (Kennedy et al., 2010). Diet diversity is somewhat lower for the rural population than the urban population, averaging 7.9 and 8.7 respectively across all survey rounds, but the national average dietary diversity score varied little over time, at approximately 8.1. Note that these scores are substantially higher than typical 24 h recall scores, as they represent diet diversity over a 7-day period. Using the community survey data, we confirmed that the number of food items available in the markets did not significantly vary over time.

A possible explanation for a lack of diversification into consumption of non-staple foods as average incomes rise would be if income growth were very unevenly distributed, thus contributing to improvements in consumption for only a small share of households. This inference does not appear to be supported by the Gini coefficients presented in Table 1.^1^ These numbers indicate that economic inequality fell slightly at the national level. Rural areas had very marginal increases in inequality while urban areas had slight decreases between 2008 and 2019. Taken together, these findings suggest there may be other explanations for the apparent lack of responsiveness of food consumption at home to rising incomes. We return to these later in the paper.

Table A4 in the Annex presents the results of Table 1 by geographical zone. The Coastal and Northern Highlands zones had higher average consumption expenditures. We refer to these as the “higher income” zones. Households in the Lake, Central, Southern Highlands, and Southern zones had lower consumption than the Tanzanian average and we refer to these as the “lower income” zones. In Table A4, we find expected results given these geographical definitions. The higher income zones had higher urban shares of population, and households in these zones spent a lower share of their income on food, but a larger share of their food budget on food away from home. Over the survey years, the higher and lower income zones each made up about half of our sample.

Next, we zoom in to examine food consumption patterns in finer detail. Table 2 presents the share of households that reported consuming each of the 12 food groups plus FAFH in the week preceding the survey, and the average amounts of foods consumed at home in kilograms per AE annually (these values were extrapolated from the amount consumed weekly). The survey was run year-round throughout the country, thereby accounting for seasonal variations in consumption at the aggregate level. As noted above, given the increase in consumption expenditure in Table 1, we would expect to see a diversification of the diet away from staples. While we did not find evidence of this trend with respect to diet diversity scores, it may be apparent in terms of the amount of each food group consumed.

**Table 2:** Share of households consuming food groups (%), and average consumption of foods (kg/AE/year), by survey round 2008–2019.

	A. Share (%) consuming					B. Amount consumed (kg/year/AE)				
	2008	2010	2012	2014	2019	2008	2010	2012	2014	2019
Cereals	93.6	94.0	91.7	96.5	89.5	179.0	158.2	154.8	164.7	138.1
Starches	71.8	72.8	70.6	73.7	67.2	95.9	70.9	71.9	71.7	72.5
Veg	94.2	95.0	92.3	96.2	92.3	42.2	44.5	47.6	55.4	46.8
Fruit	49.6	57.9	53.9	60.8	55.8	21.1	21.0	20.8	26.2	23.2
Pulses/nuts	84.9	81.6	80.6	83.2	75.7	34.2	26.9	30.1	28.6	24.8
Meat	50.6	54.8	51.8	56.4	49.7	11.9	12.0	11.7	12.9	11.0
Eggs	16.5	16.0	15.7	19.6	17.0	0.5	0.5	0.5	0.6	0.5
Fish	58.9	64.5	64.5	71.2	74.1	8.9	8.8	9.0	9.8	11.3
Dairy	32.5	34.0	32.3	34.8	30.0	13.3	14.2	15.2	16.8	12.3
Sugars	68.8	72.1	69.9	75.2	69.9	11.2	9.9	9.7	10.1	8.4
Oil	77.9	83.1	81.6	89.1	85.7	6.1	6.8	6.7	7.8	6.9
Misc. Bev.	97.4	97.4	95.2	97.9	93.2	21.0	17.4	13.3	11.8	15.4
FAFH	47.4	54.5	55.4	54.4	56.2	420.0	574.6	653.4	597.4	693.7

*Results are nationally representative with the use of survey weights. Panel B averages are unconditional. FAFH measured in estimated kcals per AE per day.

Panel A does not reveal any clear trends in the share of households consuming the different food groups over time. However, in Panel B, we find that the amount of some food groups consumed has changed. On average, comparing 2019 with 2008, households in the sample consumed less pulses (−27%), sugar (−29%), cereals and starches (−25%), and more fish (+27%). In Panel B, we also estimate the number of kcals consumed away from home, using the method from Moltedo et al. (2018) and find that these increased by 65% from 2008 to 2019. It therefore appears that there is a substitution effect, with FAFH taking the place of some of the staples, sugar, oils, and pulses, previously eaten at home, but with the increase in kcals derived from FAFH very likely exceeding the foods eaten at home that they have replaced.

Table A4 and A5 in the Annex present Table 2 results by zone. We find geographic variation for some food groups, often aligning with the foods produced in each area. For example, the Central zone is a major maize growing area and we find that households there consumed the most cereals but less starches and less fruit, likely because those items are not produced there. The Northern Highlands consumed more meat and dairy compared to the other zones, and less fish, corresponding to their status as an important livestock zone. The Coastal zone consumed the most fish, followed by the Lake and Southern zones, which have access to Lake Victoria and Lake Malawi, respectively, and also had higher than average fish consumption.

Low levels of dietary diversification over time and wide variations in diet by agro-ecological zone might indicate that most food eaten at home originates from subsistence production. Survey data do not support this hypothesis. We analyze the origin of food eaten at home (self-produced, purchased, or gifted) and variations over survey years and zones in Table 3. A large majority of food eaten at home (as a share of value) was purchased in all survey years, rising from 64% at the national level in 2008 to 74% in 2019. As expected, urban households purchased at least 90% of food consumed at home in all survey rounds. More strikingly, the share of food eaten at home purchased by rural households climbed steeply, from 54.2% in 2008 to 67.5% in 2019.

**Table 3:** Share of purchased food in value of food eaten at home by zone, 2008–2019.

	Tanzania	Rural	Urban	Coastal	N. Highlands	Lake	S. Highlands	Central	Southern
**2008**	64.2	54.2	92.9	80.5	81.3	58.0	58.5	53.1	53.6
**2010**	68.6	59.3	90.4	83.5	78.6	67.3	63.8	54.5	55.7
**2012**	68.3	57.8	92.5	85.1	75.2	62.0	61.3	59.2	56.8
**2014**	72.6	61.7	94.6	89.6	81.6	63.8	68.7	60.9	66.9
**2019**	74.1	67.5	90.2	86.8	80.0	69.7	63.3	67.5	68.0

*Results are nationally representative with the use of survey weights.

The deepening integration of rural dwellers into food markets could suggest that incipient commercialization of subsistence agriculture is taking place, but likely also reflects increasing levels of engagement in non-farm wage work and associated access to cash incomes. The average number of household members with non-farm employment is presented in Table A7. We see a clear increase, particularly in rural households, of participation in non-farm employment. Over 40% of households still did not have any members with non-farm employment in 2019, but this is down from 60% in 2008.

The trends outlined above are reflected at the zone level. Households in the higher income Coastal and Northern Highland zones purchased a larger share of their food than the average Tanzanian household. Households in the four lower income zones purchased a lower share of their food than the national average, but this share increased more rapidly than for urban households or households in the higher income zones. This pattern aligns with our finding that households in the lower income zones also increased their expenditure on FAFH most rapidly.

Together these findings indicate that consumption patterns among households in more rural and less economically developed areas of Tanzania are transforming more rapidly than those in more urban/economically developed zones. These changes might be expected to be associated with consumption of a more diverse and nutritious diet, as seen in numerous low- and middle-income countries (Clements and Si, 2018; Mayén et al., 2014). However, we have not seen evidence for this trend thus far. We return to this point later.

### 3.2 Exploratory pathways

**Fig. 1:**
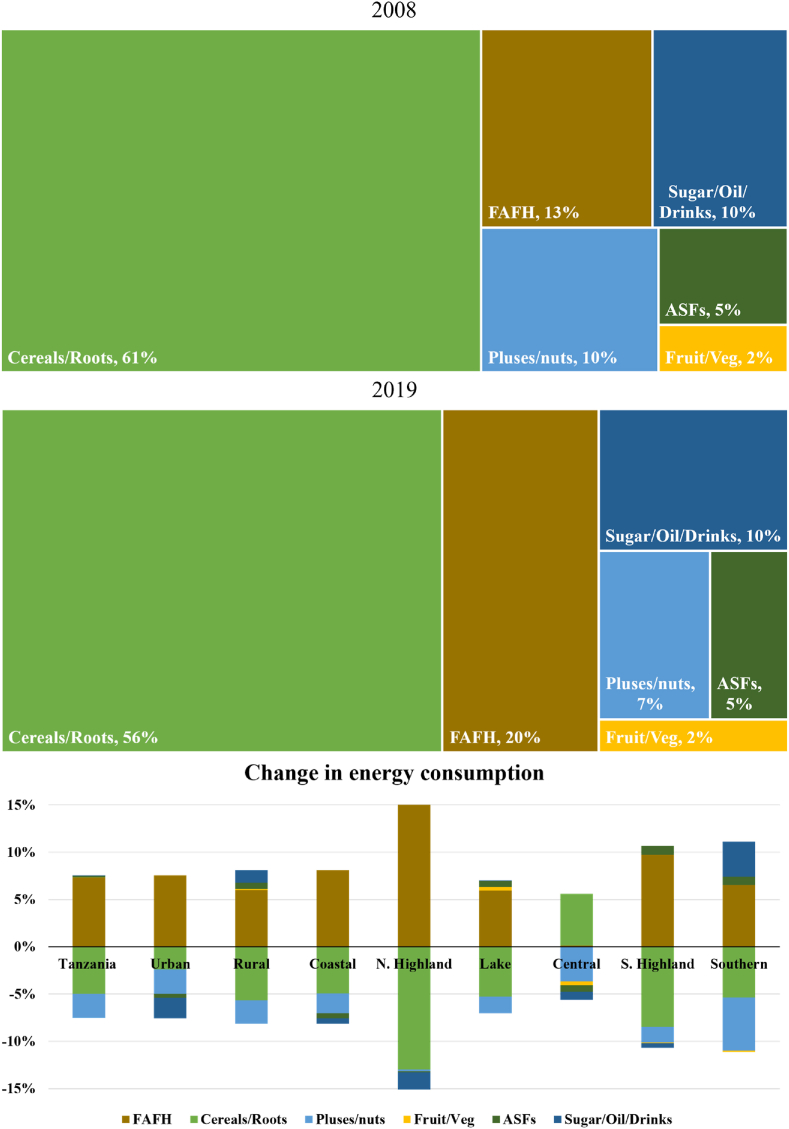
Average share of dietary energy by food group and changes in their contribution to energy consumption by zone, 2008 and 2019.

Source: Author's calculations

To better understand the average Tanzanian diet, we move to Fig. 1. The two upper panels in Fig. 1 compare the share of average dietary energy consumption originating from six aggregated food groups in the first and last survey years (2008 and 2019). The lower panel presents changes in energy consumption from each food group between the two surveys, by geographical zone.

The share of energy derived from staples (cereals/roots) and pulses/nuts consumed at home decreased everywhere between survey years, except in the lower income Central zone (which saw a slight increase in energy consumption from staples), falling by an average of 5 and 2.5 percentage points, respectively, nationally. Conversely, the share of energy obtained from FAFH increased by more than 7 percentage points nationally. This finding is consistent with other recent literature from Tanzania, which found increases in income over time leading to more food being eaten away from home (Cockx et al., 2018; Sauer et al., 2021). The higher income Northern Highlands saw the largest increase in share of energy originating from FAFH and the lower income Central zone experienced no change in FAFH. Aside from a substitution of FAFH for staples, changes in the share of energy derived from other food groups were minimal.

**Fig. 2:**
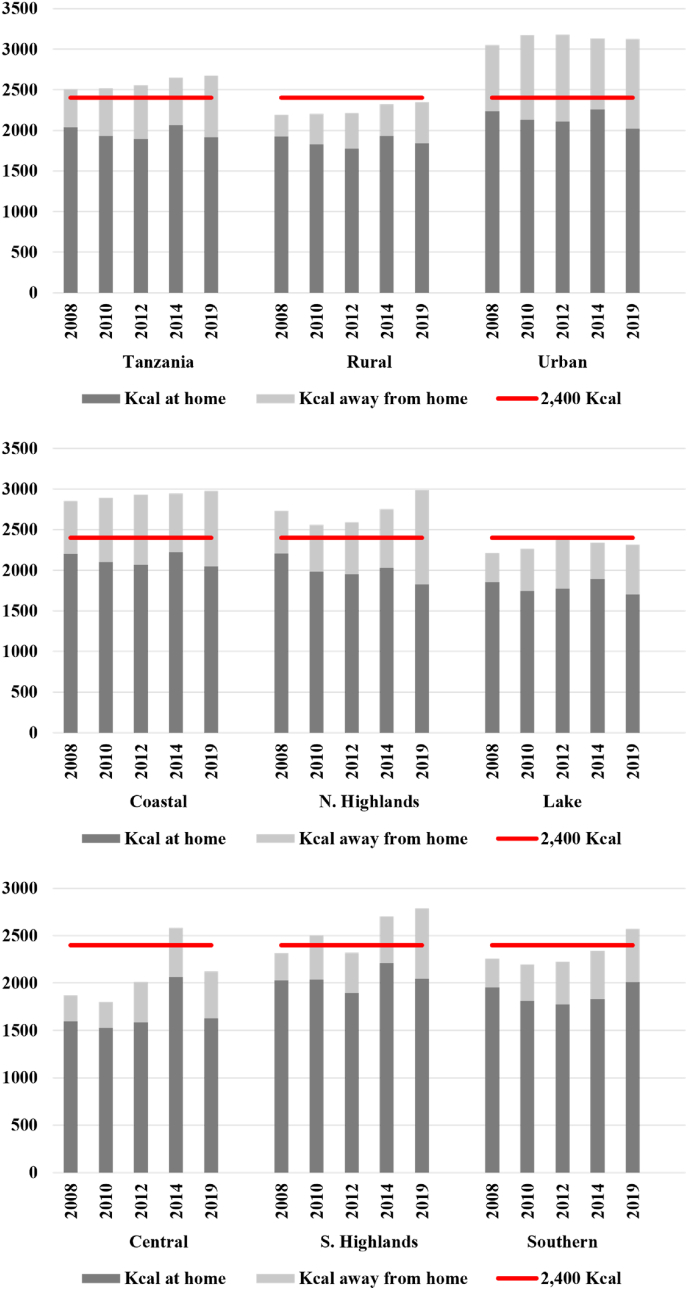
Total energy consumption by zone, 2008–2019.

Source: Author's calculations

Next, we assess changes over time in absolute consumption of energy, expressed in kcals, derived from food eaten at home and FAFH, by zone (Fig. 2). Energy consumed at home is represented by dark gray bars and the estimated energy derived from FAFH is depicted as light gray bars. The horizontal red line denotes the 2400 kcals that should be the average daily energy consumed per AE. We find that in all zones, the average Tanzanian adult did not meet their daily energy requirements without FAFH. However, even after accounting for energy from FAFH, on average, rural individuals did not meet their daily energy requirements in any survey year, and households in the four “lower income” zones fell short on average in several survey rounds. In contrast, estimated daily energy consumption in urban areas exceeded energy requirements by around 30% in 2019, with most of this surplus attributable to energy derived from FAFH. Thus, for many rural dwellers FAFH makes an important contribution to meeting energy needs, whereas the amounts of FAFH consumed in urban areas likely drive excessive energy consumption.

**Fig. 3:**
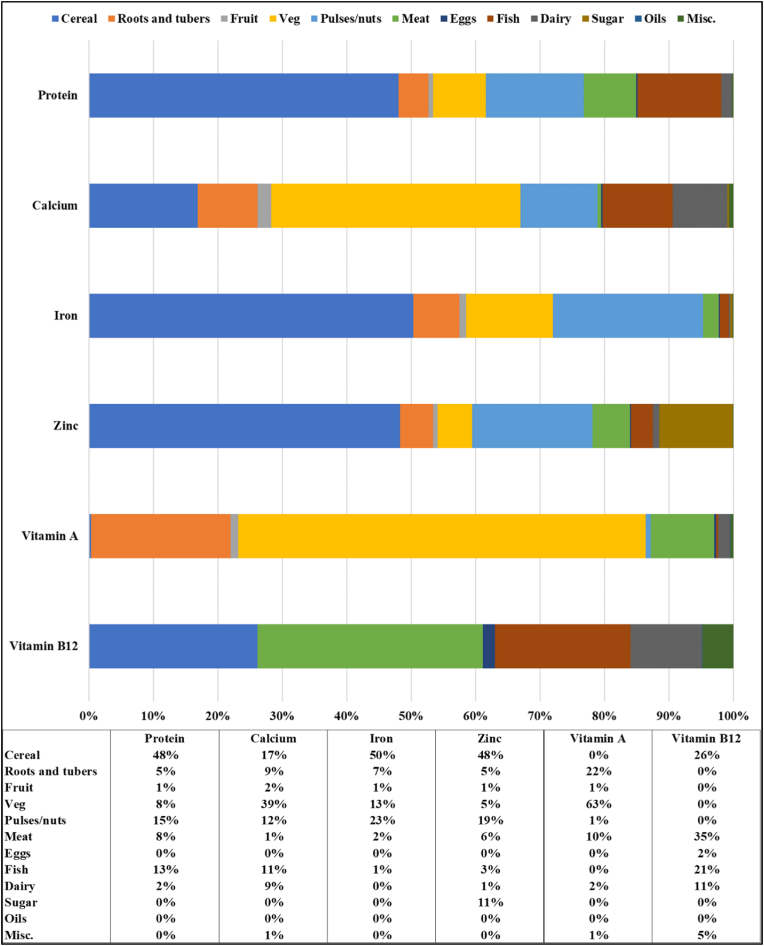
Average share of selected nutrients in food eaten at home derived from major food groups.

Source: Authors' calculations. All five survey rounds are pooled for this figure as there was little change over time.

Having evaluated the source and amount of energy consumed in the Tanzanian diet, we now present estimates of the shares of selected nutrients (protein, calcium, iron, zinc, vitamin A, and vitamin B12) derived from the twelve food groups that comprise food eaten at home (cereals, roots and tubers, fruit, vegetables, pulses/nuts, meat, eggs, fish, dairy, sugar, oils, miscellaneous) (Fig. 3). We find that these shares changed minimally across survey years; i.e., very little dietary diversification took place from 2008 to 2019. Because there was very little change over this period, Fig. 3 is constructed using pooled data from all five survey years.

Staple foods made a large contribution to the consumption of most nutrients. This finding points to the very low diversity of the Tanzanian diet. Staples were the primary source for protein, iron, and zinc, with cereals alone accounting for around half of the consumption of all three nutrients, and around one-quarter of vitamin B12, while roots and tubers contributed close to one quarter of vitamin A. Vegetables were the main source of vitamin A (63%) and calcium (39%). Pulses and nuts accounted for between 12% and 23% of protein, calcium, iron and zinc. Animal-sourced foods (meat, fish, eggs, and dairy combined) contributed a small share of nutrient consumption, apart from vitamin B12 (69%), protein (23%) and calcium (20%). Fruits contributed very little to nutrient consumption (2% or less for all nutrients). It must be noted that we only take into account key macro- and micronutrients. However, the importance of fiber and phytonutrients in the diet should not be underestimated. Fruit and vegetables are among the main sources of these healthy compounds and a lack of consumption is therefore worrisome.

**Fig. A1:**
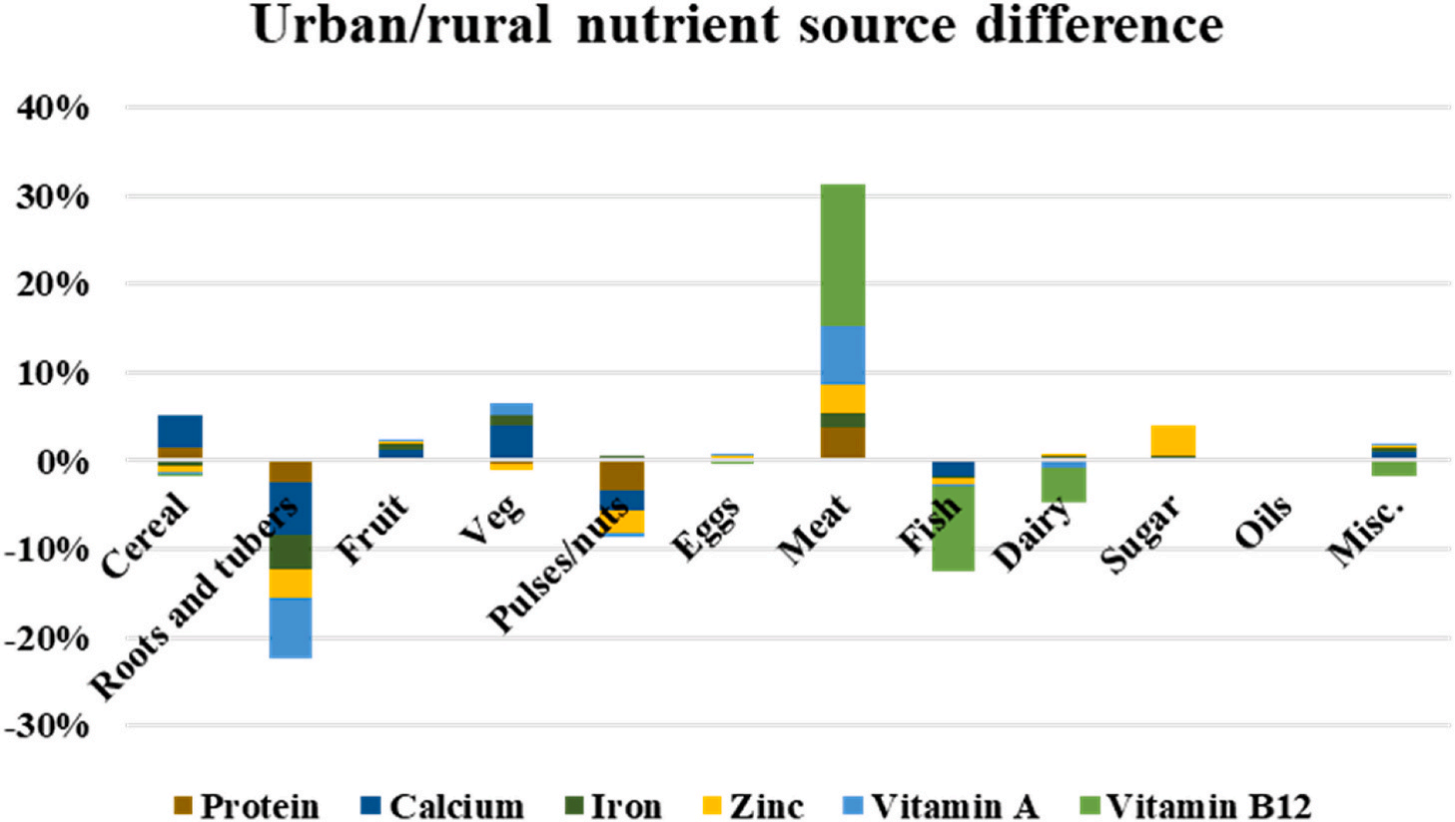
Average rural/urban difference in share of selected nutrients in food eaten at home derived from major food groups .

Figure A1 in the Annex presents rural/urban differences in the availability of these nutrients. Negative (positive) values indicate that rural (urban) households receive more of a given nutrient from a given food source. We find that urban households consumed more of their nutrients from fruit, vegetables, and meat than rural households. Roots and tubers were important sources of all nutrients except vitamin B12 for rural households. Additionally, fish and dairy were important sources of vitamin B12 in rural households. Important B-vitamins are generally most readily available in animal-sourced foods.

**Fig. 4:**
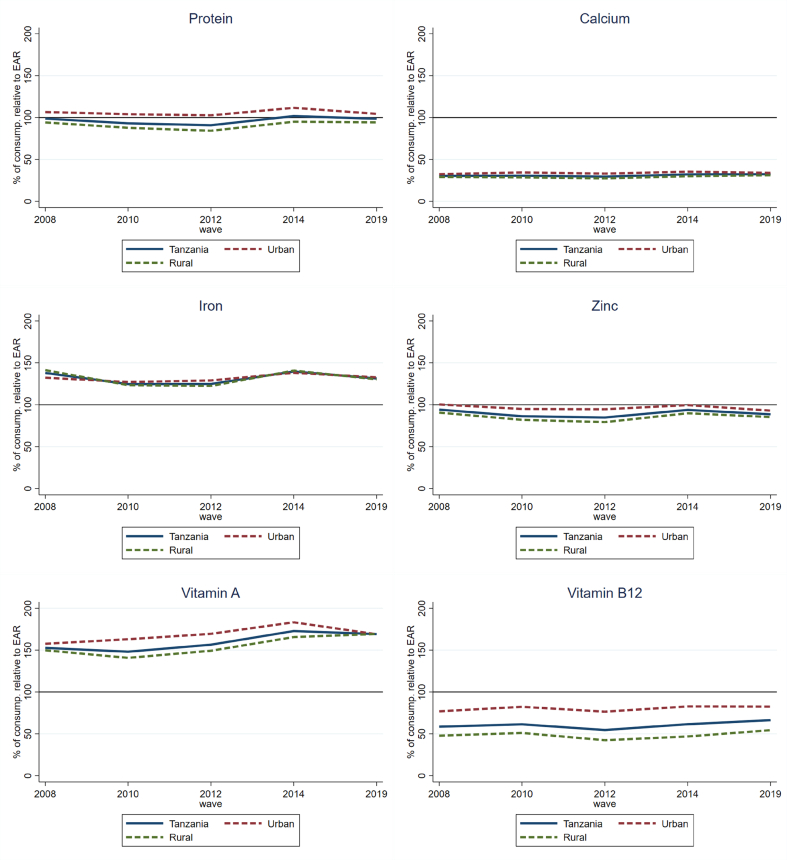
Consumption of selected nutrients relative to EARs (%) by rural/urban, 2008–2019.

Source: Authors' calculations.

Next, following Ameye et al. (2021), we interpret the nutrition implications of the consumption patterns described with reference to their contributions to EARs for each nutrient, by zone. An EAR of 100 indicates perfectly adequate nutrient consumption, while values below (above) 100 indicate the degree to which the average household member under (over) consumes that nutrient. This measure indicates the depth of nutrient (in)adequacy, making it more meaningful than a binary indicator of nutrient adequacy (presented in the Annex in Table A7). For example, the binary nutrient adequacy measure suggests that about one-third to one-half of the sample in each zone is protein deficient, whereas EARs show that on average, individuals fulfil well over 80% of their protein requirements. These results are presented in Fig. 4. The results are only for foods consumed at home, as the nutrient composition of FAFH is less reliable.

Fig. 4 shows that Tanzanians had high average levels of vitamin A and iron adequacy (>100 for national, rural, and urban areas, in all survey years), moderate protein and zinc inadequacy, and high vitamin B12 and calcium inadequacy. About half of the iron consumed was derived from cereals (mainly maize). However, non-heme iron obtained from plant-source foods is less bio-available than heme iron from ASFs, so iron adequacy may be lower than Fig. 4 suggests. Moderate levels of protein and zinc inadequacy likely reflect high levels of consumption of cereals, which were the main source of both these nutrients for households in our sample. In contrast, high levels of vitamin B12 and calcium inadequacy reflect low levels of ASF consumption, as ASF are particularly rich sources of these nutrients (Adesogan et al., 2020).

Comparing nutrient adequacy for rural and urban households, we find minimal differences between the two groups. The nutrients with the largest gap between rural and urban consumption (with urban being higher) are vitamin A and vitamin B12. As noted above, rural households consumed an adequate amount of vitamin A. However, all households were, on average, deficient in vitamin B12, with the adequacy of rural households falling 30 percentage points lower than that of urban households.

**Fig. A2:**
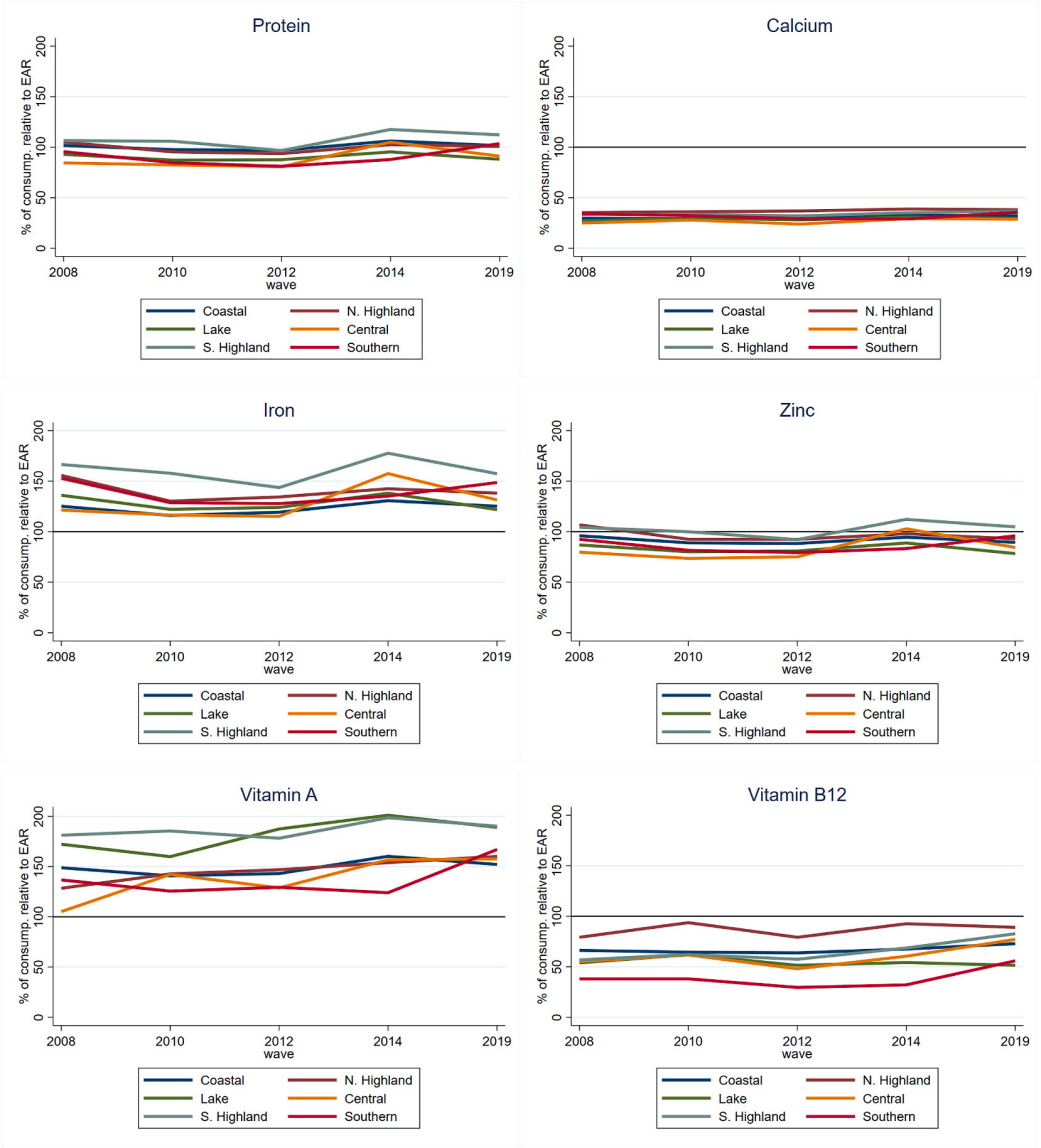
Consumption of selected nutrients relative to EARs (%) by geographical zone, 2008–2019..

Reviewing these patterns by zone (Figure A2 in the Annex), the lower income Southern Highlands had higher levels of protein, iron, zinc, and vitamin A adequacy than other zones, while two other lower income zones (Central and Southern) had generally lower levels of nutrient adequacy. Studying Table A6, higher rates of nutrient adequacy in the Southern Highlands may be due to their high consumption of cereals but also higher consumption of animal-source foods than other lower income zones. Adequacy of iron, vitamin A, and vitamin B12 consumption vary most between zones, with less variability for protein, calcium (highly inadequate in all zones), and zinc.

**Fig. 5:**
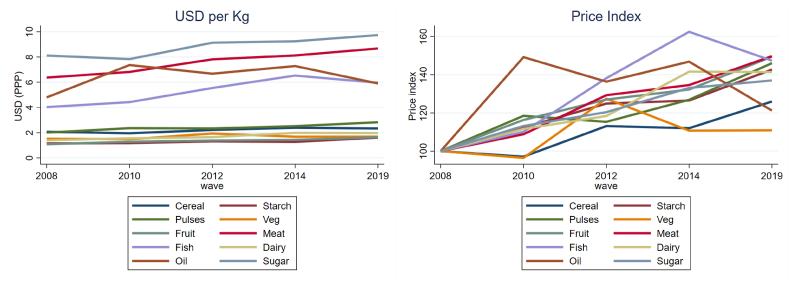
Food prices per kilogram and food price index, 2008–2019.

Source: Authors' calculations.

A crucial finding from Fig. 4 is that nutrient adequacy did not improve significantly over time. This finding is consistent with the lack of diversification in foods eaten at home across survey years described above. We hypothesize that diets might not have diversified as expected with rising incomes to include larger quantities of more nutritious foods if the real price of nutritious foods also increased. Other studies from low-, middle-, and high-income countries have found that nutritious foods are becoming more expensive (Ameye et al., 2021; Dizon and Herforth, 2018; Wiggins et al., 2015). To test this hypothesis, we evaluate the changing cost of nutrient-rich foods by calculating the average price in each survey year in USD/kg at constant 2011 PPP prices, and a price index, where the price in 2008 is equal to 100 (Fig. 5).

**Fig. A3:**
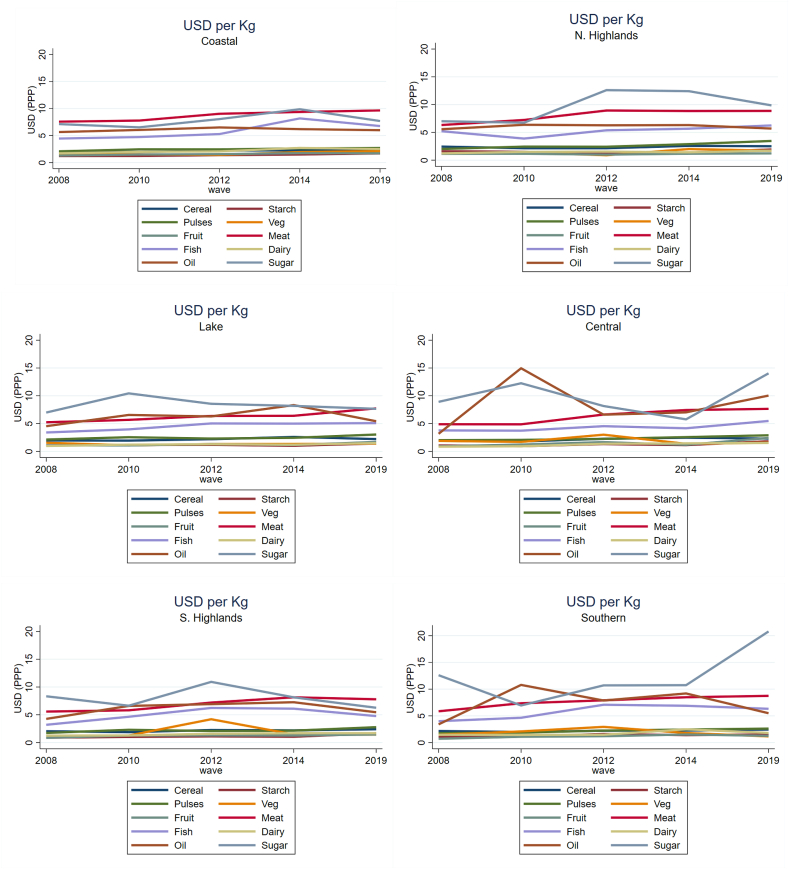
Food prices per kilogram by zone, 2008–2019..

**Fig. A5:**
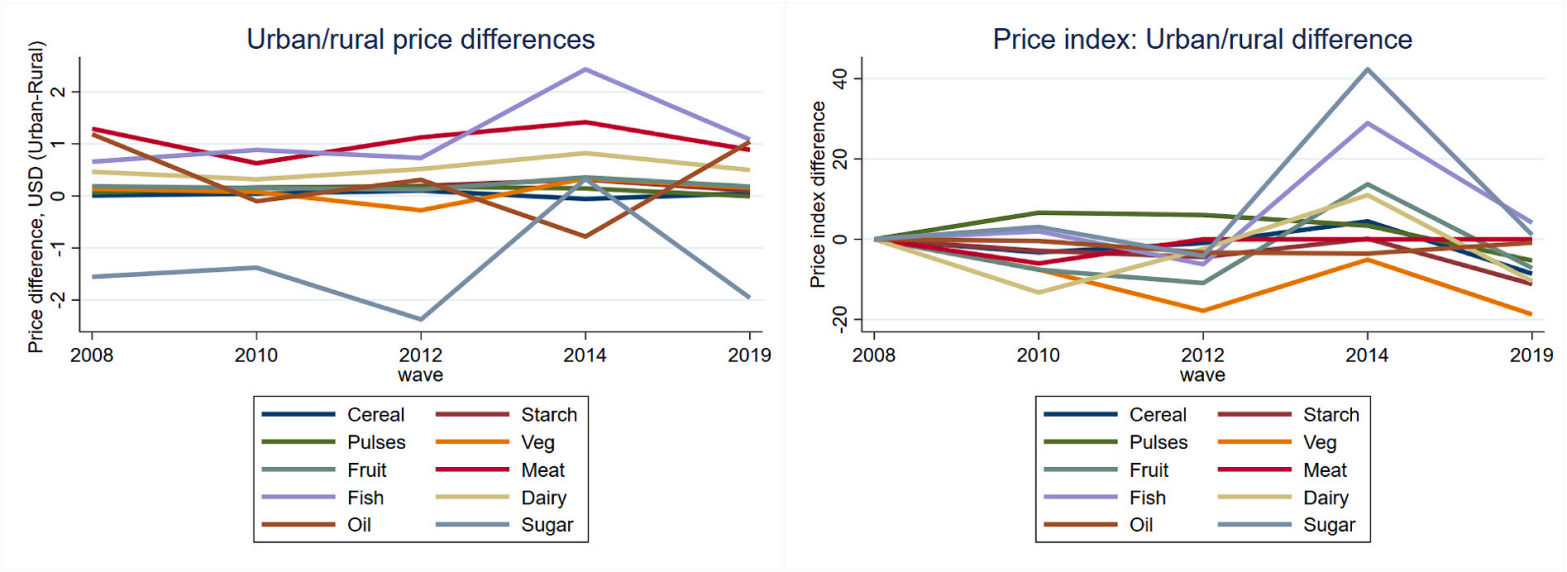
Urban/rural food prices per kilogram and price index, 2008–2019 .

Roots, vegetables, and fruit were the cheapest food groups per kilogram. Dairy (mainly milk) was by far the cheapest ASF, expressed per kilogram basis (USD1.97/kg in 2019). Sugars were the most expensive food group at USD 9.74/kg, followed meats (USD 8.68/kg). All food groups became more expensive between 2008 and 2019, but to varying degrees. Most food groups (roots, pulses, fruit, meat, fish, and dairy) increased by around 40–50% over this period. Cereal and vegetable prices increased the least, but still rose by 10–25% per kilogram. Figures A.3 to A.5 in the Annex present these results by zones and rural/urban location. The right panel of Figure A5 presents the rural/urban difference between price indexes. The negative values indicate that the prices of these foods increased more for rural households than for urban ones.

**Fig. 6:**
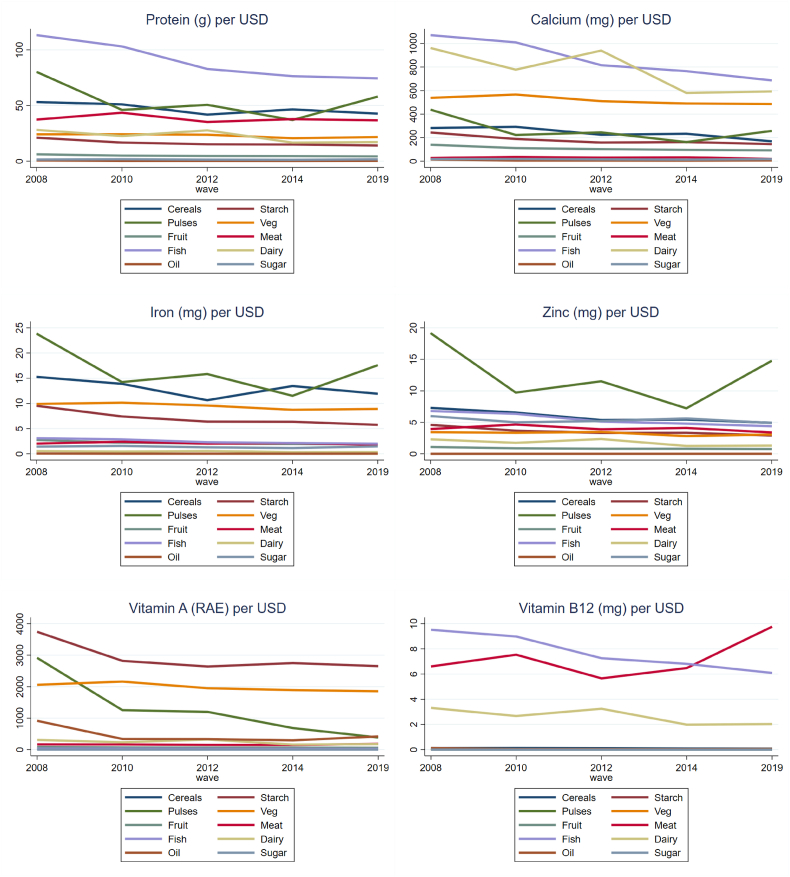
Amount of nutrients obtained per USD, by food group, 2008–2019.

Source: Authors' calculations.

We next calculate the cost of individual nutrients obtained from the food groups. These results allow us to identify the most affordable sources of nutrients and changes in the affordability of those nutrients over time. Fig. 6 presents the amount of each nutrient that one USD could buy in each survey year. We find that fish was the most affordable source of protein, calcium, and vitamin B12 in four out of five years. Despite their high affordability in terms of price per unit of many nutrients, fish and meat contribute to only a small share of nutrients in the Tanzanian diet (as seen in Fig. 3), likely reflecting their high price per kg relative to foods such as cereals, pulses, and vegetables. Pulses/nuts were the most affordable sources of zinc and iron, while root vegetables were the most affordable source of vitamin A in our study.

We also find that the quantity of nutrients that could be purchased with one USD declined somewhat across survey rounds for most foods. This pattern aligns with the price index results presented in Fig. 5. In other words, the cost of meeting nutritional requirements increased over time. At the national level, the amount of calcium derived from one USD's worth of the least expensive source (fish) fell by 56% from 2008 to 2019. The amount of protein and vitamin A obtained from the least expensive sources (fish and starches) fell by a similar amount, with one USD purchasing 52% and 41% less of each nutrient in 2019 than in 2008. The amount of iron and zinc purchased by one USD decreased by 35% and 30% respectively. The amount of vitamin B12 derived from the least expensive source increased by only 2%. This was the only nutrient for which the most affordable source changed between 2008 and 2019, shifting from fish in 2008 to meat in 2019.

**Fig. A6:**
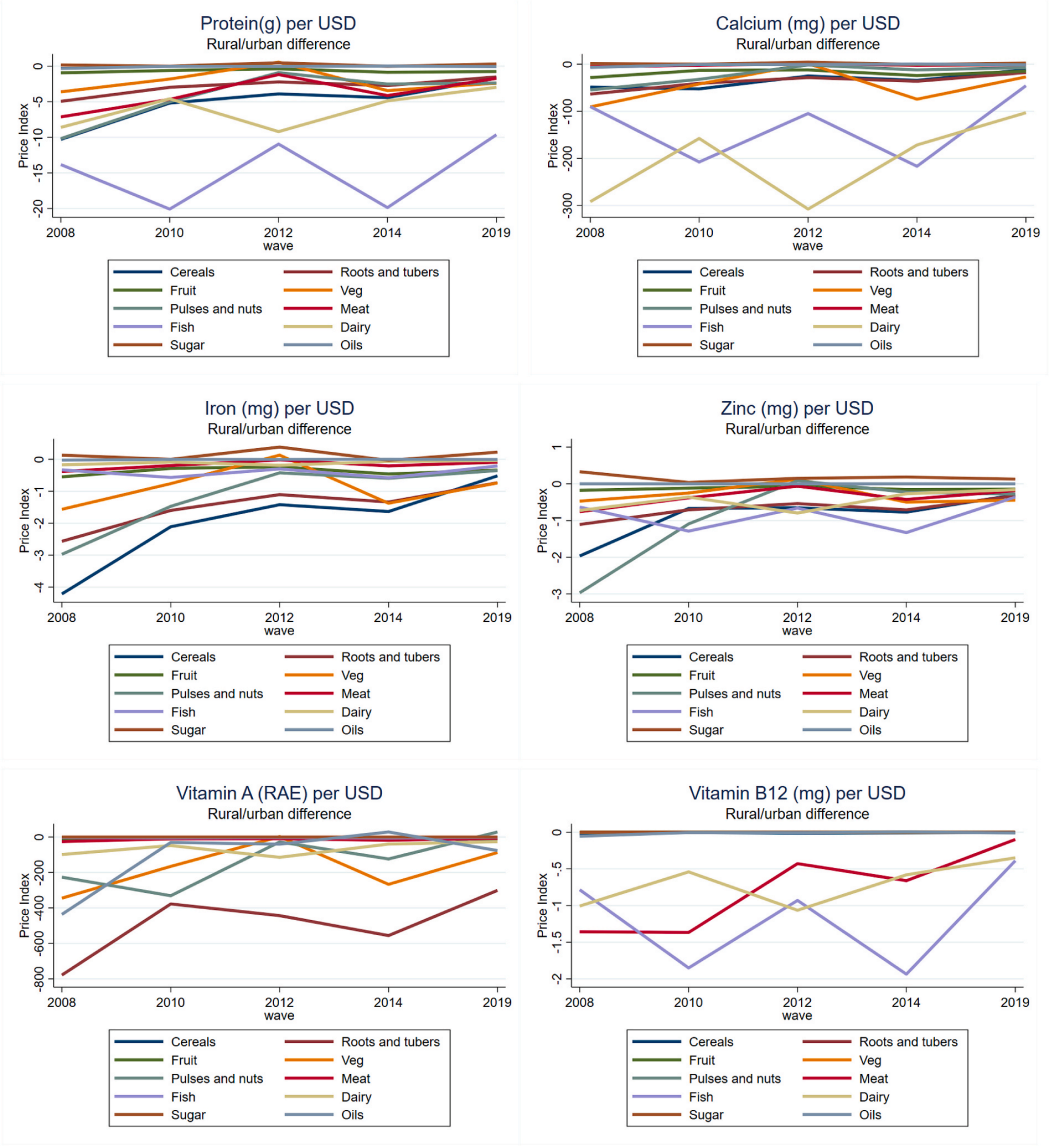
Rural/urban difference in the amount of nutrients from animal source food obtained per USD, 2008–2019 .

Results from Fig. 6 are presented in Tables A9 to A14 and Figure A6 Figure A6, by zone, year, and rural/urban location. Nutrients were more expensive in rural areas than in urban during this period, but this gap appears to have narrowed over time. In a context where most foods, particularly non-staples, are purchased, these trends are likely to complicate efforts to promote nutritious diets, as foods and the nutrients they provide become increasingly expensive in real terms.

Lastly, we examine the relationship between household nutrient adequacy and cost of nutrients, using ordinary least squares regressions, pooling results from all survey rounds. Results are presented in Table A15. Our dependent variable is the nutrient adequacy of the household (values from Fig. 4) and the independent variables of interest are the cost of nutrient per USD (values from Fig. 6). Additional control variables are location (rural/urban), household size, share of food budget spent on FAFH, and participation in non-farm employment by the household head. We also use fixed effects for zone, survey month, and survey year.

As expected, we find negative associations between the cost of nutrients derived from cereals, vegetables, and meat and levels of iron and zinc adequacy. This suggests that the more affordable these nutrients from these sources are, the less iron and zinc deficiency is found, on average. We also find a strong and negative relationship between nutrient adequacy and the share of the household food budget that is spent on FAFH. This is expected as the adequacy index only includes foods consumed at home, not food away from home. Therefore, typically, the more a household spends on FAFH, the less data we have on the total nutrients consumed.

Lastly, we find a positive relationship between nutrient adequacy and participation of the household head in non-farm employment for all dependent variables, except iron. Sauer et al. (2021) also studied consumption in Tanzania and found that non-farm employment increased FAFH for both men and women. In sum, from our results and the literature it appears that non-farm employment can both increase nutrient adequacy (for from food consumed at home) and increase FAFH consumption. This may seem contradictory, but is evidence of two possible opposing mechanisms. While non-farm employment can improve nutrition at home, it is also associated with increases in consumption of FAFH which is likely less healthy, therefore potentially offsetting the nutritional gains.

## 4 Discussion and conclusions

### 4.1 Discussion

This study contributes to a growing contemporary literature on diet quality and accessibility in the Global South (e.g., Bai et al., 2021; Mahrt et al., 2019; Masters et al., 2018; Schneider et al., 2021). Many such studies are global in scope, although more recent country-specific work is also taking place. Our paper helps fill this information gap for one of Africa's largest and fastest growing economies, Tanzania, for the period 2008–2019. We study changes in: (1) Dietary composition; (2) Adequacy of consumption of key nutrients relative to EARs; (3) The cost of foods and the micronutrients derived from them.

Our approach combines multiple data sources to increase the accuracy of estimates of nutrient consumption and prices. This approach shows that nutritious foods are becoming more expensive in Tanzania, and that the country is reaching a critical juncture in its nutrition transition. Increasing incomes have not been associated with improvements in the quantity or diversity of non-staple foods consumed at home, whereas consumption of less healthy FAFH has increased dramatically, and at a faster rate for rural households than for urban ones.

Rural-urban differences are stark. People in rural areas experienced lower nutrient adequacy and faced more rapidly rising prices for key nutritious foods than those in urban areas. On average, members of rural households did not meet their entire energy needs. For these individuals, FAFH played an important role in meeting the most basic of dietary requirements. In contrast, while average energy consumption from food eaten at home was also inadequate for urban dwellers, high levels of FAFH consumption mean that urban energy consumption exceeded average daily requirements by around 30%, contributing to overconsumption of energy.

Even in lower-income rural zones, households purchased most of the food they consumed at home. The share of purchased food increased steadily over the period studied, to reach 70% and 78% on average for rural and national households, respectively. Deepening integration into markets coupled with rising real incomes might be expected to stimulate consumption of more diverse nutritious foods (fruit, vegetables, and ASF) if access to markets increases the variety of nutritious foods available for consumption (Sibhatu et al., 2015). Contrary to expectations, this has not happened. However, a recent study suggests that a slow transformation toward larger, more commercialized farms has begun (Wineman et al., 2020). One of the indicators noted by these authors is the decrease in the share of the population employed in agriculture while the sector still grows. This has resulted in more of the population relying on markets for food as opposed to subsistence farming.

A possible explanation for the lack of diversification or increase in the quantity of more nutritious foods eaten at home is that the real price of these foods has increased over time. This pattern is particularly evident for rural households, which faced larger increases in the prices of most foods than urban ones. This was especially the case for nutrient dense ASFs such as fish and meat, which are among the most important sources of vitamin B12. Notably, fish, the most affordable source of protein, calcium, and vitamin B12, faced a larger relative increase in price than any other food group, contributing to these nutrients becoming more expensive over time.

As a result, we find no evidence of improvements in the nutritional adequacy of the average Tanzanian diet over the survey period. The steeply rising real prices of nutritious foods suggests that their supply may not have kept pace with rising demand. Increasing prices of such foods might be expected to induce a supply response from commercializing producers and supply chains. This trend has been observed in other rapidly developing African countries such as Ethiopia (Minten et al., 2016, 2020) and Nigeria (Liverpool-Tasie et al., 2021). This observation raises the question of why there has apparently been little supply side response to rising incomes in Tanzania. Part of the explanation may relate to relative prices. It is not possible to reliably track changes in the price of FAFH using the datasets analyzed, but the uptick in their consumption suggests that they may have become cheaper, either in real terms, or relative to more nutritious foods eaten at home. International food price inflation over the survey period may also have influenced domestic food prices in Tanzania.

We also recognize another possible explanation, which is consumer preferences: as processed foods have gained popularity in Tanzania, these nutrient-poor foods may be preferred due to taste or convenience. FAFH does not require preparation, lowering the opportunity costs of consumption relative to home-cooked food. For example, firewood is the most common cooking fuel (64% for full sample across all years, and 88% for the rural population) but takes time to collect, therefore increasing the opportunity cost of meals cooked at home.

In sum, our findings indicate that in Tanzania rising incomes and better nutrition from food consumed at home do not go hand in hand. Diet diversification has occurred, but only in the direction of FAFH. Rather than diversification away from staples into more nutritious unprocessed foods being consumed at home, the accelerating consumption of food eaten outside the home appears to have bypassed dietary diversification at home almost entirely. Our data does not let us comment on the diversity of FAFH, only that the amount spent on FAFH increased dramatically from 2008 to 2019.

### 4.2 Policy and research implications

The scenario outlined above places Tanzania's food system at a crucial juncture and highlights the need for nutrition-sensitive policies. Popkin and Ng (2022) contend that LMIC do not need to follow the same path as high-income countries that currently experience persistently high level of consumption of ultra-processed foods (stage 4 of Popkin's nutrition transition), and the disease burden resulting from their consumption. They note that lower- and middle-income countries can avoid this steep increase in consumption of processed foods if policymakers step in. We find that the continued rapid growth of FAFH (much of which is comprised of ultra-processed foods or other foods of poor nutritional value) is likely to result in negative nutritional outcomes for the Tanzanian population, requiring urgent policy responses. These policies will need to consider both the demand- and the supply-sides, as well as the supply chains and food environments that link producers and consumers (Ruel et al., 2018).

From the supply side, actions are needed to promote production of nutritious foods and ensure that they are both affordable and physically available to consumers. Without concurrent increases in the production of these foods and the supply chain actors and infrastructure needed to distribute them to consumers, prices will continue to increase and may ultimately reduce levels of consumption and lower micronutrient intakes. For example, our analysis shows that fish, particularly in its dried product form, is the most affordable source of several key micronutrients, but as it becomes more expensive, this advantage is shrinking. However, promoting increased production of dried fish from capture fisheries that are already subject to heavy fishing pressure will be difficult. Here, a variety of approaches, including ensuring that fisheries are governed in ways that support more sustainable management practices, seeking to reduce loss and waste in fisheries supply chains, or promoting imports for fish from other countries may be required.

Production of livestock and livestock products in predominantly pastoralist systems (in the case of cattle) or backyard production (in the case of poultry) may also be difficult to intensify, possibly pointing to opportunities for establishing more specialized dairy herds or intensive forms of feedlot based chicken production. Such enterprises may require elements of an enabling environment – e.g., infrastructure, utilities, foreign investment, expertise, and imported inputs – that are currently difficult to access. Further, Tanzania's local governments charge a ‘produce cess’, a levy of up to 5% of the farmgate price. These rates are set locally and provide funds to local governments, but vary widely between regions, to the extent that some farmers move to areas with lower cess rates. Reforming this system could help promote production of non-staple foods (Nyange et al., 2015).

From the demand side, other studies have found that demand for healthy foods such as fruit and vegetables in Tanzania has not increased (Abdulai and Aubert, 2004; Ochieng et al., 2018). Demand remains low partially due to cultural perceptions of these foods being signs of poverty, or food for livestock (Kansiime et al., 2018). While we also find that per capita consumption of fruit and vegetables changed little over the survey period, it is not clear whether this pattern reflects a lack of demand *per se*, or changes in relative prices favoring the consumption of FAFH. Interventions in Tanzania to promote fruit and vegetable consumption through homegardens have found mixed evidence. Blakstad et al. (2021) found positive effects of a homestead food production program on women's dietary diversity in the rural areas of the Coastal zone. Depenbusch et al. (2021) found a positive increase of a homegarden promotion project on vegetable production, but no impact on diets in the Northern Highland and Coastal zones. In either case, information campaigns to promote consumption of these products might help improve the nutritional status, though such campaigns will be more effective if accompanied by efforts to enhance the supply and distribution of these foods.

Finally, the importance and rapidly rising consumption of FAFH demands closer attention in future research to exactly which items are being eaten away from home, and at what prices. Moreover, more information is required on the distribution of FAFH within households as well. If women or children are less active outside the home than adult men, they would appear less likely to access these food items. A study by Ochieng et al. (2017) found that women and children in Tanzania had less diverse diets compared to the men in their households due to men consuming more FAFH. The authors suggest that households could have healthier diets if men consumed less FAFH and put that expenditure towards food consumed at home. On the other hand, if women's work outside the home increases as incomes rise, consumption of FAFH or more processed foods may also increase due to their increasing opportunity costs of time, and more limited time available for preparation of foods at home (Ameye and De Weerdt, 2020; Celnik et al., 2012; Garawi et al., 2014).

As such, improving the granularity of data on FAFH, its relationship to the total amount of processed food consumed by households, and the food environments that supply it, should be a priority in the design of future household living standards measurement surveys worldwide (de Brauw and Herskowitz, 2021), as the varieties, ingredients, preparation, marketing, and consumption of FAFH and processed foods will be crucial sites for potential demand-side interventions such as nutrition education, taxes, and regulation.

## Declaration of competing interest

The authors declare that they have no known competing financial interests or personal relationships that could have appeared to influence the work reported in this paper.

## Figures and Tables

**Table A1:** Total number of households, by geographical zone and survey round 2008–2019.

	Tanzania	Urban	Rural	Coastal	N. Highlands	Lake	Central	S. Highlands	Southern
**2008**	3222	1185	2037	1310	206	718	142	364	482
**2010**	3902	1286	2616	1521	236	961	175	419	590
**2012**	4991	1787	3204	1836	281	1408	227	490	749
**2014**	3344	1361	1983	1304	216	1048	128	344	304
**2019**	1180	501	679	472	41	326	59	119	163

**Table A2:** Share of urban households, by geographical zone and survey round 2008–2019.

	Tanzania	Coastal	N. Highlands	Lake	Central	S. Highlands	Southern
**2008**	26.2	49.0	30.1	18.0	13.8	17.5	19.1
**2010**	30.9	51.2	29.0	24.6	21.6	23.4	24.6
**2012**	31.7	60.1	28.6	16.6	14.7	29.2	22.5
**2014**	33.6	61.7	30.1	20.6	17.2	26.9	21.3
**2019**	30.9	60.0	28.8	16.0	14.3	25.3	22.4

Results are nationally representative with the use of survey weights.

**Table A3:** Food groups and their corresponding weights from food diaries

Food Group	Weight	Sub categories	Food Group	Weight	Sub categories
**Rice (paddy)**	1			0.167	*Pumpkin leaves*
**Rice (husked)**	1			0.167	*Cabbage*
**Maize (green, cob)**	1			0.167	*Cassava leaf*
**Maize (grain)**	1	*Kernels*	**Canned, dried and wild vegetables**	1	*Mushroom*
		*Dried*	**any vegetable other than cabbage and pumpkin**	0.5	*Cauliflower*
**Maize (flour)**	1			0.5	*Mushroom*
**Millet and sorghum (grain)**	0.5	*Millet*	**Pumpkin**	1	
	0.5	*Sorghum*	**Ripe bananas**	1	
**Millet and sorghum (flour)**	0.5	*Millet*	**Oranges, tangerines..**	0.2	*Orange*
	0.5	*Sorghum*		0.2	*Tangerine*
**Wheat, barley grain and other cereals**	1	*Wheat flour*		0.2	*Lemon*
**Bread**	1	*Bread, white*		0.2	*Lime*
				0.2	*Pineapple*
**Buns and biscuits**	1	*Biscuits*	**Fruit**	0.125	*Papaya*
**Macaroni, spaghetti**	1	*Macaroni*		0.125	*Avocado*
**Other cereal products**				0.125	*Mango*
**Cassava fresh**	1			0.125	*Durian*
**Cassava dry/flour**	1			0.125	*Melons*
**Sweet potatoes**	1			0.125	*Cucumber*
**Yams/cocoyams**	1	*Yams*		0.125	*Apple*
**Irish potatoes**	1			0.125	*Pear*
**Cooking bananas, plantains**	1		**Sugarcane**	1	
**Other starches**	1	*Pumpkin*	**Goat meat**	0.4	*Goat meat*
**Sugar**	1			0.2	*Sheep meat*
**Honey, syrups, jams, marmalade, jellies, canned fruits**	1	*Honey*		0.4	*Meat broth*
**Sweets (candy, chocolate)**	1	*Chocolate*	**Beef including minced sausage**	0.94	*Beef*
**Peas, beans, lentils and other pulses**	0.25	*Green beans*		0.03	*Beef intestines*
	0.25	*Mung Bean*		0.03	*Beef liver*
	0.25	*Hyacinth beans*	**Bacon/Pork**	1	
	0.25	*Lentils*	**Chicken and other poultry**	0.8	*Chicken*
**Groundnuts in shell/shelled**	1			0.2	*Duck*
**Coconuts (mature/immature)**	1		**Wild birds, edible insects**	1	*Termites*
**Cashew, almonds and other nuts**	0.5	*Cashew nut*			*Crickets*
	0.5	*Almond*	**Other domestic/wild meat products - rabbit**	1	*Rabbit*
**Seeds and products from nuts/seeds (excl. cooking oil)**	1	*Sunflower*	**Eggs**	1	*Chicken eggs*
**Onions, tomatoes, carrots, green pepper**	0.125	*Okra*	**Fresh fish and seafood (including dagaa)**	0.5	*Fresh Fish*
	0.125	*Green pepper*		0.5	*Fresh Seafood*
	0.125	*Onions*	**Dried/fish and seafood**	0.5	*Dried Fish*
	0.125	*Tomatoes*		0.5	*Dried Seafood*
	0.125	*Carrots*	**Fresh milk**	1	
	0.125	*Eggplant*	**Yoghurt**	1	*Yoghurt*
	0.125	*Zucchini*	**Milk powder**	1	
	0.125	*Spices, leaves, flowers*	**Cooking oil**	1	*Oil*
**Spinach, cabbage and other leaves**	0.167	*Spinach*	**Butter, margarine, ghee and other fat products**	0.5	*Ghee*
	0.167	*Pea leaves*		0.5	*Margarine brand*
	0.167	*Sweet Potato leaves*			

**Table A4:** Household consumption expenditure, food as a share of consumption, dietary diversity, and consumption inequality

**Consumption, USD (PPP)**						
	**Coastal**	**N. Highlands**	**Lake**	**Central**	**S. Highlands**	**Southern**
**2008**	1806	1602	1096	902	1104	1068
**2010**	1896	1552	1255	966	1259	1137
**2012**	2370	1806	1470	1107	1503	1283
**2014**	2330	2029	1366	1379	1503	1411
**2019**	2268	2426	1159	1095	1717	1584
**Food expenditure as share of consumption (%)**						
	**Coastal**	**N. Highlands**	**Lake**	**Central**	**S. Highlands**	**Southern**
**2008**	72.7	74.1	77.8	84.8	80.5	82.6
**2010**	71.1	73.6	75.7	78.7	78.8	78.3
**2012**	69.5	73.3	77.4	79.1	77.1	78.2
**2014**	68.2	73.8	76.1	72.7	76.4	76.0
**2019**	66.8	70.3	77.7	76.8	71.8	74.5
**Food eaten at home as share of consumption (%)**						
	**Coastal**	**N. Highlands**	**Lake**	**Central**	**S. Highlands**	**Southern**
**2008**	61.4	65.0	71.2	77.8	74.3	77.0
**2010**	56.7	62.8	65.4	71.3	69.1	70.6
**2012**	52.4	60.0	64.3	70.0	67.1	69.6
**2014**	53.6	58.6	66.6	64.3	67.1	65.9
**2019**	50.6	53.0	66.8	66.8	57.3	63.0
**Food away from home expenditure as share of consumption (%)**						
	**Coastal**	**N. Highlands**	**Lake**	**Central**	**S. Highlands**	**Southern**
**2008**	11.2	9.0	6.5	6.9	6.1	5.5
**2010**	14.5	10.8	10.3	7.4	9.5	7.7
**2012**	17.0	13.2	13.1	9.2	9.9	8.6
**2014**	14.6	15.2	9.4	8.3	9.3	10.2
**2019**	16.2	17.2	10.2	10.0	14.5	11.5
**Diet diversity score**						
	**Coastal**	**N. Highlands**	**Lake**	**Central**	**S. Highlands**	**Southern**
**2008**	8.6	9.2	7.8	7.0	7.7	7.3
**2010**	8.6	9.1	8.1	7.7	8.2	7.7
**2012**	8.5	8.6	7.8	7.0	8.1	7.2
**2014**	9.1	8.9	8.3	8.0	8.5	7.6
**2019**	8.5	6.8	7.8	6.8	8.8	8.1
**Gini coefficient**						
	**Coastal**	**N. Highlands**	**Lake**	**Central**	**S. Highlands**	**Southern**
**2008**	0.41	0.37	0.39	0.35	0.35	0.34
**2010**	0.40	0.35	0.38	0.37	0.35	0.36
**2012**	0.39	0.37	0.39	0.39	0.38	0.36
**2014**	0.37	0.38	0.38	0.39	0.37	0.39
**2019**	0.38	0.44	0.37	0.40	0.34	0.37

Results are nationally representative with the use of survey weights.

**Table A5:** Share of households consuming food groups, by survey round 2008–2019.

	Coastal					N. Highlands					Lake				
	2008	2010	2012	2014	2019	2008	2010	2012	2014	2019	2008	2010	2012	2014	2019
**Cereal**	95.1	94.1	92.4	97.0	91.5	97.9	97.7	92.4	94.9	75.6	90.5	91.6	88.9	95.9	89.6
**Starch**	78.2	76.4	73.7	76.1	75.0	74.1	75.8	69.2	69.1	50.9	79.1	75.3	77.4	80.8	70.8
**Veg**	92.2	91.8	90.4	96.3	89.9	94.3	95.2	89.6	91.7	77.5	93.1	95.1	92.4	96.0	96.3
**Fruit**	63.1	67.2	68.0	74.3	69.1	64.1	57.0	57.2	66.8	44.2	48.4	56.6	49.5	56.0	46.4
**Pulse/nuts**	87.9	87.4	84.9	87.6	83.4	84.7	80.5	80.4	76.9	54.0	80.1	74.6	74.5	79.9	72.3
**Meat**	54.4	54.8	58.2	62.9	52.9	69.6	74.9	67.0	69.4	42.4	49.1	52.9	45.7	51.7	43.1
**Egg**	21.6	18.9	20.7	28.4	25.8	23.7	23.6	20.6	22.7	10.3	14.7	11.7	10.0	13.4	6.0
**Fish**	70.7	71.3	71.1	80.6	76.6	52.8	46.0	45.2	55.5	53.8	54.6	70.7	72.5	74.0	79.4
**Dairy**	31.0	29.8	30.5	35.2	28.8	70.8	78.6	69.0	69.4	41.0	34.5	35.9	32.7	33.4	31.5
**Sugar**	87.7	87.6	85.6	89.1	83.9	95.7	93.4	88.5	89.8	70.7	58.0	62.5	58.9	64.6	56.7
**Oil**	82.1	85.0	82.4	90.2	85.4	94.7	92.2	87.9	91.6	77.5	76.6	82.2	82.3	89.6	87.5
**Misc. Bev.**	96.9	95.5	92.7	97.7	91.6	98.5	96.8	92.8	94.3	81.4	98.0	97.9	96.3	98.3	96.6
	**Central**					**S. Highlands**					**Southern**				
	**2008**	**2010**	**2012**	**2014**	**2019**	**2008**	**2010**	**2012**	**2014**	**2019**	**2008**	**2010**	**2012**	**2014**	**2019**
**Cereal**	98.5	100.0	97.1	99.6	88.7	94.8	96.1	95.8	99.0	96.4	90.9	91.3	88.1	92.6	86.0
**Starch**	25.8	37.8	31.2	41.3	36.4	63.7	70.8	67.2	71.5	61.3	81.1	83.8	74.5	74.0	79.0
**Veg**	98.5	100.0	96.8	98.2	94.5	97.4	96.8	95.3	98.7	94.4	94.5	95.0	91.7	94.4	92.6
**Fruit**	21.4	48.8	29.3	45.9	30.6	40.3	56.6	56.0	56.9	65.6	46.9	50.2	42.2	49.4	67.2
**Pulse/nuts**	88.1	84.0	77.8	84.4	64.8	86.1	84.3	86.4	88.8	84.6	89.8	87.8	83.8	78.3	80.6
**Meat**	46.1	59.9	50.0	64.0	43.1	52.5	57.9	57.4	58.4	73.0	31.8	36.2	34.0	32.6	42.0
**Egg**	10.0	14.5	11.8	15.7	4.9	15.1	19.0	21.0	21.0	38.6	11.9	15.4	12.2	14.0	15.4
**Fish**	47.3	43.9	47.0	47.9	60.3	52.7	53.3	52.6	62.5	72.1	68.7	74.1	65.0	78.1	80.1
**Dairy**	32.1	32.3	33.6	40.1	21.8	27.1	31.2	29.4	30.7	39.2	6.7	7.7	6.6	10.4	12.2
**Sugar**	58.7	65.5	56.3	74.6	59.1	60.8	65.8	65.3	70.4	73.1	58.4	66.8	65.6	66.3	79.1
**Oil**	79.4	85.5	73.9	91.7	87.5	79.9	87.6	89.6	90.1	89.3	53.5	66.1	64.8	78.2	80.3
**Misc. Bev.**	96.7	100.0	97.9	99.6	94.7	98.0	97.3	97.4	99.4	94.8	95.3	99.0	94.9	96.6	92.7

Results are nationally representative with the use of survey weights.

**Table A6:** Consumption amount (kg/year/AE), by survey round 2008–2019.

	Coastal					N. Highlands					Lake				
	2008	2010	2012	2014	2019	2008	2010	2012	2014	2019	2008	2010	2012	2014	2019
**Cereal**	175.0	165.4	163.5	162.4	149.7	165.5	138.7	138.0	142.7	103.3	161.3	138.1	135.5	155.9	126.7
**Starch**	84.6	62.3	59.4	65.8	77.4	87.8	69.7	74.2	66.2	95.7	117.4	86.0	92.4	91.8	77.3
**Fruit**	31.6	27.4	27.8	31.8	29.4	22.3	17.3	25.5	32.2	26.6	15.5	18.2	17.3	23.1	16.3
**Veg**	49.4	47.6	56.3	66.9	49.5	39.3	46.3	51.4	57.5	44.9	30.5	35.9	37.9	43.9	37.4
**Pulse/nut**	44.2	35.1	38.8	37.9	34.5	26.9	18.2	23.7	21.2	21.2	26.2	21.3	23.0	23.0	18.8
**Meat**	13.7	12.5	13.7	14.6	12.7	17.4	16.4	14.6	16.7	12.2	11.4	11.5	9.7	11.3	8.0
**Eggs**	0.7	0.5	0.7	1.0	0.8	0.8	0.6	0.6	0.8	0.2	0.4	0.4	0.3	0.4	0.2
**Fish**	12.1	11.5	10.9	13.1	13.1	4.3	5.1	4.6	6.1	4.2	9.3	9.1	10.9	9.7	12.1
**Dairy**	12.2	11.5	12.6	15.4	14.4	33.5	39.5	40.2	45.5	18.7	14.2	15.5	15.0	16.3	10.1
**Sugar**	16.1	13.0	12.7	12.3	10.4	18.0	14.4	14.1	14.4	9.0	9.0	8.7	8.2	8.6	5.9
**Oil**	7.3	8.3	7.8	9.2	8.5	10.4	10.1	8.7	10.9	7.4	5.0	5.9	5.8	6.7	5.8
**Misc. Bev.**	22.6	17.4	15.5	16.3	23.2	15.2	17.9	14.7	13.3	28.4	13.4	11.7	7.9	8.8	5.9
	**Central**					**S. Highlands**					**Southern**				
	**2008**	**2010**	**2012**	**2014**	**2019**	**2008**	**2010**	**2012**	**2014**	**2019**	**2008**	**2010**	**2012**	**2014**	**2019**
**Cereal**	238.4	199.2	193.5	197.2	133.7	213.4	184.3	176.9	196.2	169.6	157.0	154.8	148.5	147.4	133.1
**Starch**	17.9	17.2	19.3	24.6	24.0	84.5	59.4	58.7	67.6	50.2	140.8	99.7	94.7	66.2	92.8
**Fruit**	8.6	14.2	10.0	13.3	11.3	20.5	24.9	22.0	28.0	24.5	25.5	18.5	15.6	22.2	34.1
**Veg**	42.3	46.7	36.6	48.4	64.2	53.6	54.7	55.9	60.9	50.2	49.0	48.9	49.9	57.3	54.5
**Pulse/nut**	28.8	24.0	24.6	25.3	16.4	30.8	25.9	30.6	28.6	21.7	53.2	38.2	38.9	30.1	32.1
**Meat**	9.3	12.5	13.4	14.1	10.3	11.2	13.0	13.3	13.9	16.5	8.2	7.4	6.8	7.2	7.7
**Eggs**	0.3	0.3	0.4	0.6	0.1	0.5	0.6	0.7	0.6	0.9	0.3	0.5	0.4	0.5	0.6
**Fish**	3.8	4.0	4.2	3.6	5.1	7.2	7.3	5.7	7.8	12.5	10.0	10.3	9.4	11.6	13.0
**Dairy**	13.3	11.5	17.8	17.0	10.7	8.8	10.6	12.5	12.7	15.3	3.1	4.1	3.2	4.0	5.5
**Sugar**	7.5	7.3	6.5	10.0	5.7	9.0	8.4	7.8	8.5	11.2	7.3	8.1	7.5	7.9	8.8
**Oil**	5.4	6.1	5.0	7.9	6.0	6.5	6.7	7.8	7.6	6.7	3.6	4.5	4.3	5.4	6.9
**Misc. Bev.**	28.5	23.3	19.4	8.2	14.1	35.1	28.1	20.5	13.8	17.4	19.1	16.3	8.8	7.6	13.5

Results are nationally representative with the use of survey weights.

**Table A7:** Average number of HH members with non-farm employment by zone, 2008–2019.

	Tanzania	Rural	Urban	Coastal	N. Highlands	Lake	S. Highlands	Central	Southern
**2008**	0.41	0.15	1.12	0.76	0.46	0.33	0.14	0.24	0.24
**2010**	0.50	0.24	1.10	0.81	0.45	0.48	0.26	0.33	0.30
**2012**	0.55	0.26	1.19	0.93	0.54	0.44	0.20	0.45	0.34
**2014**	0.61	0.28	1.26	1.01	0.63	0.53	0.29	0.31	0.31
**2019**	0.65	0.39	1.23	1.04	0.69	0.41	0.33	0.75	0.48

Results are nationally representative with the use of survey weights.

**Table A8:** Share (%) of AEs with adequate consumption of nutrients, by zone over time

	Coastal					N. Highlands					Lake				
	2008	2010	2012	2014	2019	2008	2010	2012	2014	2019	2008	2010	2012	2014	2019
**Protein**	57.9	50.0	53.4	62.6	56.4	54.2	42.4	46.8	51.5	43.3	40.7	38.5	36.7	46.2	39.1
**Calcium**	0.2	1.1	0.4	0.5	1.4	0.9	0.5	0.0	2.5	0.0	0.0	0.6	0.2	1.2	0.3
**Iron**	76.5	66.7	70.7	78.0	69.0	81.0	70.5	71.7	79.4	54.5	65.8	60.5	61.1	71.8	65.1
**Zinc**	55.5	44.9	49.5	55.3	54.1	58.7	37.6	43.9	47.9	40.2	35.7	32.6	32.9	41.1	33.5
**Vit A**	57.9	56.8	58.1	68.5	59.7	52.8	54.4	60.2	66.3	45.8	47.3	49.6	52.2	60.7	51.9
**Vit B12**	30.7	29.5	29.0	32.4	35.8	32.4	43.4	32.8	41.7	34.1	22.0	25.2	19.4	23.0	15.2
	**Central**					**S. Highlands**					**Southern**				
	**2008**	**2010**	**2012**	**2014**	**2019**	**2008**	**2010**	**2012**	**2014**	**2019**	**2008**	**2010**	**2012**	**2014**	**2019**
**Protein**	39.0	34.2	30.6	52.1	46.8	56.2	58.0	48.0	62.4	65.3	45.5	35.8	31.4	38.2	54.8
**Calcium**	0.6	0.0	0.4	0.6	0.0	0.5	0.2	0.2	0.8	1.2	0.2	0.0	0.1	0.0	0.6
**Iron**	50.0	52.9	53.0	75.7	67.3	78.2	78.5	75.4	84.1	85.3	73.0	63.9	63.4	64.8	70.2
**Zinc**	35.4	28.9	27.6	52.6	42.5	55.4	52.3	45.0	59.9	62.6	44.3	35.0	31.9	37.4	46.7
**Vit A**	32.5	52.8	33.5	47.6	56.5	60.4	65.8	64.5	69.1	61.8	48.7	50.4	50.1	46.8	49.3
**Vit B12**	22.6	29.5	15.2	23.7	29.6	21.8	25.4	23.7	30.6	45.6	11.0	11.7	8.5	12.2	22.5

Results are nationally representative with the use of survey weights.

**Table A9:** Protein (g) per USD

	Coastal					N. Highlands					Lake				
	2008	2010	2012	2014	2019	2008	2010	2012	2014	2019	2008	2010	2012	2014	2019
**Cereal**	42.9	44.7	36.1	41.4	39.7	39.1	41.9	47.2	38.0	38.8	75.0	54.7	42.5	49.8	45.4
**Starch**	18.1	15.8	13.5	12.0	13.2	13.9	11.3	14.6	13.0	11.7	21.3	17.7	15.6	17.8	16.4
**Sugar**	1.4	1.8	1.6	1.0	1.6	1.6	1.7	0.9	0.7	1.0	1.5	1.1	1.1	1.2	1.6
**Pulses/nuts**	74.7	40.9	49.3	36.6	54.9	71.4	41.8	60.2	30.9	54.7	88.7	41.9	40.4	38.8	60.8
**Veg**	25.0	22.2	24.2	17.1	17.5	36.7	23.2	53.0	18.9	18.3	25.0	30.7	27.0	25.4	25.2
**Fruit**	5.7	4.8	4.5	3.9	3.8	4.9	5.0	6.1	4.9	4.8	5.8	5.5	4.8	5.1	4.8
**Meat**	30.7	41.1	34.9	34.0	34.7	53.7	45.9	31.8	34.5	36.0	37.4	42.9	38.4	39.9	39.2
**Egg**	16.3	13.0	11.6	11.7	12.8	16.2	12.8	12.9	13.9	11.9	16.1	13.3	12.6	14.3	12.5
**Fish**	**96.7**	**98.9**	**78.9**	**51.2**	**63.7**	**76.5**	**108.2**	**81.8**	**78.6**	**66.1**	**137.9**	**109.4**	**84.6**	**90.1**	**80.9**
**Dairy**	25.7	18.3	19.0	13.5	14.6	24.5	17.7	17.9	19.1	15.6	32.3	23.0	21.0	22.2	19.3
**Oil**	0.4	0.3	0.2	0.2	0.3	0.3	0.3	0.2	0.2	0.3	0.8	0.3	0.3	0.2	0.3
**Misc. Bev.**	1.4	1.0	0.8	0.7	1.1	1.2	0.9	0.8	0.7	1.1	2.1	0.9	0.7	1.1	1.3
	**Central**					**S. Highlands**					**Southern**				
	**2008**	**2010**	**2012**	**2014**	**2019**	**2008**	**2010**	**2012**	**2014**	**2019**	**2008**	**2010**	**2012**	**2014**	**2019**
**Cereal**	68.3	67.3	46.5	51.6	51.3	52.8	57.8	41.3	49.6	44.7	52.1	56.3	49.3	48.5	40.1
**Starch**	47.2	19.5	19.8	13.8	12.3	29.3	20.1	15.8	18.5	14.1	22.1	17.4	18.1	14.4	15.2
**Sugar**	1.0	0.7	1.1	1.6	2.7	1.5	1.7	0.8	1.3	1.4	1.2	1.5	0.9	1.4	0.8
**Pulses/nuts**	78.9	47.6	52.8	35.9	53.2	86.9	72.4	71.4	50.9	60.8	94.5	52.5	54.2	34.9	57.7
**Veg**	20.1	21.9	13.9	25.2	23.0	27.4	25.2	18.4	22.6	22.1	22.8	20.7	15.7	18.4	30.6
**Fruit**	6.7	4.9	3.4	5.2	2.5	7.2	4.7	4.9	5.3	4.4	8.7	5.5	5.2	4.2	5.1
**Meat**	40.9	50.5	43.6	46.2	39.0	48.5	71.1	34.8	46.9	39.9	44.8	37.9	32.5	36.3	35.9
**Egg**	20.9	17.1	14.7	14.1	10.6	13.8	13.4	13.6	13.3	14.4	12.6	13.7	11.7	11.7	11.2
**Fish**	**117.4**	**115.1**	**94.6**	**119.4**	**77.6**	**125.1**	**88.3**	67.2	**69.8**	**111.4**	**106.8**	**89.3**	**63.0**	**63.2**	**69.0**
**Dairy**	34.6	49.7	20.6	18.5	19.6	30.4	21.3	**91.3**	16.9	18.8	33.6	32.2	23.0	12.7	18.4
**Oil**	0.8	0.1	0.2	0.2	0.1	1.2	0.3	0.2	0.2	0.3	1.1	0.1	0.2	0.1	0.3
**Misc. Bev.**	1.8	1.5	1.0	1.1	0.9	1.6	1.4	0.9	1.0	0.7	1.4	1.4	0.9	0.6	0.9

Results are nationally representative with the use of survey weights.

**Table A10:** Calcium (mg) per USD

	Coastal					N. Highlands					Lake				
	2008	2010	2012	2014	2019	2008	2010	2012	2014	2019	2008	2010	2012	2014	2019
**Cereal**	212.4	225.4	182.8	187.4	151.9	154.5	219.0	347.1	171.0	154.4	351.7	336.6	223.0	261.1	178.6
**Starch**	204.8	176.2	139.7	123.6	134.5	141.5	114.5	137.0	133.0	120.5	247.7	203.6	169.2	202.0	172.8
**Sugar**	16.1	19.0	18.1	10.7	17.5	19.6	16.4	8.7	8.4	9.7	15.9	13.7	11.7	13.3	15.6
**Pulses/nuts**	428.6	198.5	227.8	176.2	246.7	398.1	211.9	365.4	139.2	243.7	468.2	212.3	190.8	169.5	264.7
**Veg**	580.8	503.2	506.6	406.4	441.5	646.8	512.2	663.7	496.3	380.3	611.0	682.2	588.8	596.7	540.5
**Fruit**	140.1	106.8	103.2	84.0	85.4	115.6	107.9	127.1	104.0	117.1	128.2	126.0	108.4	110.6	96.1
**Meat**	19.5	35.1	32.4	30.0	16.9	55.0	38.1	28.1	30.5	18.3	24.1	35.8	28.9	32.9	19.5
**Egg**	64.9	51.6	46.0	46.4	50.7	64.5	50.9	51.2	55.3	47.3	63.8	52.9	50.0	56.7	49.8
**Fish**	**954.0**	**989.1**	**778.6**	**490.9**	**624.1**	686.3	**1056.5**	**825.6**	**801.4**	**621.2**	**1370.4**	**1068.5**	**821.2**	**904.2**	**698.3**
**Dairy**	877.7	633.7	652.1	470.2	502.4	**847.3**	615.0	620.9	662.3	543.8	1117.0	802.4	730.4	773.2	666.0
**Oil**	8.5	5.5	4.8	4.4	6.2	6.1	5.5	5.2	5.2	6.6	17.4	5.4	5.8	4.1	6.7
**Misc. Bev.**	36.0	28.6	23.9	23.7	33.1	37.9	27.3	24.0	22.1	32.2	66.6	29.4	21.4	29.1	37.0
	**Central**					**S. Highlands**					**Southern**				
	**2008**	**2010**	**2012**	**2014**	**2019**	**2008**	**2010**	**2012**	**2014**	**2019**	**2008**	**2010**	**2012**	**2014**	**2019**
**Cereal**	453.1	423.7	323.8	251.6	222.0	286.8	290.9	217.6	267.1	171.8	330.2	373.5	247.2	232.0	165.8
**Starch**	724.7	231.4	168.3	146.2	132.2	302.9	229.9	163.9	194.9	139.6	243.4	196.2	190.3	160.0	159.5
**Sugar**	13.3	8.7	11.6	17.9	21.8	16.9	16.9	9.2	15.2	14.9	18.4	15.1	11.1	13.1	13.2
**Pulses/nuts**	419.5	233.9	238.8	156.6	238.7	561.7	356.9	320.6	210.8	264.6	442.7	250.8	273.3	149.1	264.2
**Veg**	470.1	574.0	373.5	550.2	541.1	528.6	596.3	533.3	547.0	496.5	525.0	566.2	417.1	430.4	657.7
**Fruit**	143.3	108.5	90.8	129.3	63.0	166.6	127.2	103.7	111.5	100.8	212.1	119.8	117.5	90.8	115.5
**Meat**	25.8	39.3	34.2	37.0	19.8	31.0	52.2	29.4	37.2	20.2	32.5	30.6	22.8	32.7	18.4
**Egg**	83.0	67.8	58.2	56.0	42.2	54.9	53.0	53.8	52.9	57.3	50.2	54.5	46.6	46.4	44.6
**Fish**	867.8	1128.5	**917.4**	**1282.1**	**692.7**	**1061.9**	**827.2**	633.2	**640.1**	**987.6**	965.2	844.9	612.3	**619.2**	**653.5**
**Dairy**	**1202.2**	**1682.7**	716.5	644.5	676.1	1035.3	743.9	**3000.7**	589.4	649.5	**1129.8**	**1087.7**	**787.8**	439.5	635.9
**Oil**	17.2	1.6	4.7	4.2	2.6	24.1	7.1	5.0	4.0	6.6	23.6	2.5	4.3	3.0	6.6
**Misc. Bev.**	47.8	36.4	27.5	28.2	29.8	43.0	38.0	26.1	25.8	25.9	39.3	33.8	24.5	20.6	29.0

Results are nationally representative with the use of survey weights.

**Table A11:** Iron (mg) per USD

	Coastal					N. Highlands					Lake				
	2008	2010	2012	2014	2019	2008	2010	2012	2014	2019	2008	2010	2012	2014	2019
**Cereal**	11.6	11.1	8.6	**11.7**	10.8	10.4	10.8	12.6	10.8	**10.7**	22.9	**15.7**	11.4	**14.6**	12.3
**Starch**	7.9	7.0	5.6	5.0	5.5	5.9	4.9	5.8	5.3	5.0	9.5	7.9	6.7	7.6	6.9
**Sugar**	1.4	1.6	1.5	0.9	1.5	1.6	1.5	0.8	0.7	0.9	1.4	1.1	1.0	1.2	1.4
**Pulses/nuts**	**22.6**	**13.1**	**15.8**	11.4	**17.4**	**22.5**	**13.3**	**17.7**	9.9	**17.3**	**25.6**	12.9	**12.8**	11.8	**17.5**
**Veg**	10.4	9.2	9.6	7.3	7.6	13.5	9.5	17.2	8.4	7.3	10.7	12.6	11.0	10.7	10.1
**Fruit**	2.7	2.1	2.0	1.7	1.7	2.3	2.2	2.5	2.2	2.4	2.5	2.5	2.2	2.2	2.0
**Meat**	1.7	2.3	2.0	1.9	1.7	3.2	2.5	1.8	2.0	1.7	2.1	2.5	2.2	2.3	1.9
**Egg**	1.6	1.2	1.1	1.1	1.2	1.5	1.2	1.2	1.3	1.1	1.5	1.3	1.2	1.4	1.2
**Fish**	2.7	2.8	2.2	1.4	1.8	2.0	3.0	2.3	2.2	1.8	3.8	3.0	2.3	2.5	2.1
**Dairy**	0.5	0.3	0.3	0.3	0.3	0.5	0.3	0.3	0.3	0.3	0.6	0.4	0.4	0.4	0.3
**Oil**	0.0	0.0	0.0	0.0	0.0	0.0	0.0	0.0	0.0	0.0	0.1	0.0	0.0	0.0	0.0
**Misc. Bev.**	0.1	0.1	0.1	0.1	0.2	1.2	0.3	0.2	0.1	0.2	3.3	0.8	0.2	0.2	0.2
	**Central**					**S. Highlands**					**Southern**				
	**2008**	**2010**	**2012**	**2014**	**2019**	**2008**	**2010**	**2012**	**2014**	**2019**	**2008**	**2010**	**2012**	**2014**	**2019**
**Cereal**	21.9	**18.9**	13.3	**12.9**	15.7	16.0	16.6	10.8	**15.1**	13.1	15.4	**17.0**	12.5	**14.9**	11.5
**Starch**	**28.0**	8.4	8.1	5.6	4.7	12.3	9.1	6.4	7.7	4.8	9.8	8.1	8.1	6.7	6.4
**Sugar**	1.1	0.7	1.0	1.5	2.1	1.4	1.5	0.8	1.3	1.3	1.4	1.3	0.9	1.2	1.0
**Pulses/nuts**	21.5	15.0	**16.0**	10.8	**16.8**	**23.9**	**21.3**	**21.3**	14.8	**17.9**	**30.1**	15.8	**17.0**	11.5	**17.9**
**Veg**	8.5	9.7	6.3	10.2	9.6	10.6	10.7	8.6	9.7	9.1	9.5	9.4	7.0	7.7	12.3
**Fruit**	3.0	2.2	1.7	2.4	1.3	3.4	2.5	2.1	2.3	2.0	4.0	2.4	2.3	1.8	2.3
**Meat**	2.3	2.8	2.4	2.4	1.9	2.5	3.5	2.0	2.5	2.0	2.3	2.1	1.7	2.0	1.7
**Egg**	2.0	1.6	1.4	1.3	1.0	1.3	1.3	1.3	1.3	1.4	1.2	1.3	1.1	1.1	1.1
**Fish**	2.9	3.2	2.6	3.4	2.1	3.3	2.4	1.8	1.9	3.0	2.9	2.4	1.7	1.8	1.9
**Dairy**	0.7	1.0	0.3	0.3	0.3	0.6	0.4	2.2	0.3	0.4	0.7	0.7	0.4	0.3	0.3
**Oil**	0.1	0.0	0.0	0.0	0.0	0.1	0.0	0.0	0.0	0.0	0.1	0.0	0.0	0.0	0.0
**Misc. Bev.**	1.2	0.3	0.1	0.1	0.2	1.2	0.9	0.1	0.1	0.2	1.2	0.3	0.1	0.1	0.2

Results are nationally representative with the use of survey weights.

**Table A12:** Zinc (mg) per USD

	Coastal					N. Highlands					Lake				
	2008	2010	2012	2014	2019	2008	2010	2012	2014	2019	2008	2010	2012	2014	2019
**Cereal**	5.4	5.6	4.4	4.6	4.5	5.7	6.3	5.8	4.6	4.7	11.3	7.2	5.6	6.0	5.1
**Starch**	3.9	3.5	3.0	2.6	2.8	2.9	2.4	3.1	2.8	2.5	4.6	3.8	3.4	4.1	3.4
**Sugar**	6.1	5.2	5.4	5.4	5.0	6.1	5.1	4.9	5.2	4.6	5.5	4.6	4.9	5.4	4.7
**Pulses/nuts**	**17.4**	**8.5**	**11.7**	**7.3**	**14.5**	**17.2**	**8.7**	**13.3**	**6.2**	**14.5**	**22.1**	**8.5**	**8.0**	**7.4**	**14.9**
**Veg**	3.5	3.1	3.6	2.3	2.3	5.9	3.3	9.6	2.4	2.7	3.3	4.4	3.9	3.5	3.7
**Fruit**	1.0	0.8	0.8	0.7	0.7	0.9	0.9	1.1	0.9	0.9	1.0	1.0	0.8	0.9	0.9
**Meat**	3.3	4.4	4.0	3.7	3.2	6.2	4.8	3.7	3.8	3.4	4.1	4.9	4.2	4.4	3.7
**Egg**	1.4	1.1	1.0	1.0	1.1	1.4	1.1	1.1	1.2	1.0	1.4	1.2	1.1	1.2	1.1
**Fish**	6.0	6.2	4.9	3.1	3.9	4.4	6.6	5.2	5.0	3.9	8.6	6.7	5.2	5.6	4.5
**Dairy**	2.1	1.4	1.4	1.1	1.2	1.9	1.2	1.3	1.3	1.1	2.5	1.6	1.4	1.5	1.3
**Oil**	0.0	0.0	0.0	0.0	0.0	0.0	0.0	0.0	0.0	0.0	0.0	0.0	0.0	0.0	0.0
**Misc. Bev.**	0.1	0.1	0.1	0.0	0.1	0.1	0.0	0.1	0.1	0.1	0.1	0.1	0.1	0.0	0.1
	**Central**					**S. Highlands**					**Southern**				
	**2008**	**2010**	**2012**	**2014**	**2019**	**2008**	**2010**	**2012**	**2014**	**2019**	**2008**	**2010**	**2012**	**2014**	**2019**
**Cereal**	9.8	9.5	6.8	**7.1**	5.7	7.2	7.1	5.4	6.1	5.5	7.3	7.1	6.3	5.4	4.9
**Starch**	11.6	4.4	4.5	3.0	2.6	6.3	4.4	3.3	4.1	2.7	4.7	3.9	4.0	3.2	3.2
**Sugar**	5.6	4.4	5.0	5.6	5.1	5.7	4.9	4.7	5.4	4.5	5.6	4.7	4.7	5.4	4.3
**Pulses/nuts**	**17.0**	**9.6**	**12.1**	6.8	**14.2**	**17.7**	**18.0**	**17.5**	**9.3**	**15.1**	**25.6**	**10.2**	**12.5**	**7.0**	**14.9**
**Veg**	2.8	2.8	1.7	3.6	3.2	4.2	3.4	2.1	3.0	3.1	3.2	2.5	1.9	2.5	4.4
**Fruit**	1.2	0.9	0.6	0.9	0.4	1.3	0.8	0.9	0.9	0.8	1.5	1.0	0.9	0.8	0.9
**Meat**	4.4	5.4	4.5	4.7	3.7	4.8	6.5	4.0	4.7	3.8	4.5	4.0	3.3	4.0	3.2
**Egg**	1.8	1.5	1.3	1.2	0.9	1.2	1.2	1.2	1.2	1.3	1.1	1.2	1.0	1.0	1.0
**Fish**	5.9	7.1	5.8	7.9	4.5	6.9	5.3	4.0	4.1	6.4	6.2	5.4	3.9	3.9	4.1
**Dairy**	2.7	4.2	1.3	1.3	1.3	2.6	1.5	9.5	1.3	1.5	2.9	2.8	1.8	1.0	1.4
**Oil**	0.0	0.0	0.0	0.0	0.0	0.0	0.0	0.0	0.0	0.0	0.0	0.0	0.0	0.0	0.0
**Misc. Bev.**	0.1	0.1	0.1	0.1	0.0	0.0	0.0	0.1	0.0	0.1	0.0	0.1	0.0	0.0	0.1

Results are nationally representative with the use of survey weights.

**Table A13:** Vitamin A (RAE) per USD

	Coastal					N. Highlands					Lake				
	2008	2010	2012	2014	2019	2008	2010	2012	2014	2019	2008	2010	2012	2014	2019
**Cereal**	10.6	9.6	8.1	14.6	15.4	12.2	16.8	15.7	13.0	16.7	16.4	16.3	12.6	17.7	16.6
**Starch**	**3016.8**	**2577.6**	**2429.7**	**2074.1**	**2237.3**	2083.9	1864.8	2521.2	**1988.6**	**1530.1**	**4268.5**	**3072.7**	**2770.5**	**3501.5**	**3269.5**
**Sugar**	0.0	0.0	0.0	0.0	0.0	0.0	0.0	0.0	0.0	0.0	0.0	0.0	0.0	0.0	0.0
**Pulses/nuts**	3386.2	1184.6	783.3	1059.0	373.4	**2850.8**	1475.8	**3414.7**	672.6	338.5	2592.0	1475.6	1108.5	730.4	338.4
**Veg**	2209.8	1933.3	1931.6	1595.3	1708.0	2332.2	**1978.9**	2417.5	1915.1	1475.5	2347.1	2587.7	2239.2	2274.1	2045.5
**Fruit**	73.2	66.2	59.7	50.2	52.4	64.6	64.2	84.5	61.0	55.1	80.1	78.1	64.4	68.4	62.7
**Meat**	158.9	151.1	138.5	133.8	172.6	166.3	156.1	134.7	132.5	174.2	199.9	194.1	182.0	171.4	207.2
**Egg**	172.5	137.1	122.3	123.4	134.9	171.4	135.5	136.2	147.0	125.7	169.8	140.7	132.9	150.8	132.5
**Fish**	26.5	27.5	21.6	13.6	17.3	19.0	29.3	22.9	22.3	17.2	38.1	29.7	22.8	25.1	19.3
**Dairy**	279.5	179.8	194.3	132.8	151.3	247.3	163.7	171.3	179.8	146.9	323.5	214.6	192.8	205.6	191.0
**Oil**	549.1	350.8	311.0	283.8	397.6	394.5	350.9	334.9	334.9	424.0	1121.6	348.6	374.3	263.5	430.6
**Misc. Bev.**	5.8	5.0	4.9	5.3	6.2	5.3	4.7	4.7	5.0	6.1	5.2	4.7	4.6	5.0	6.2
	**Central**					**S. Highlands**					**Southern**				
	**2008**	**2010**	**2012**	**2014**	**2019**	**2008**	**2010**	**2012**	**2014**	**2019**	**2008**	**2010**	**2012**	**2014**	**2019**
**Cereal**	24.4	21.6	20.3	18.2	26.2	15.8	13.7	10.8	19.7	17.1	18.1	18.0	12.9	16.6	16.3
**Starch**	**5290.9**	**3744.3**	**2399.0**	**2808.7**	**2179.1**	5014.0	**3245.8**	**3156.3**	**3171.6**	**3697.3**	**4221.7**	**2379.4**	**2574.3**	**2186.0**	**3020.5**
**Sugar**	0.0	0.0	0.0	0.0	0.0	0.0	0.0	0.0	0.0	0.0	0.0	0.0	0.0	0.0	0.0
**Pulses/nuts**	2850.6	1475.6	763.6	672.6	338.3	**6025.0**	1482.5	691.7	673.1	338.6	936.4	1475.7	1521.9	543.3	573.4
**Veg**	1835.2	2213.2	1471.6	2077.0	2009.4	2057.3	2313.8	2083.5	2146.0	1922.8	2054.4	2155.1	1642.6	1668.7	2442.6
**Fruit**	80.4	67.2	48.2	72.3	24.5	97.2	58.2	62.9	70.4	49.0	130.6	71.5	68.1	63.9	61.5
**Meat**	206.6	216.8	166.3	162.2	187.1	183.8	196.0	156.1	157.6	196.2	160.3	135.2	127.7	121.5	150.8
**Egg**	220.8	180.3	154.7	149.0	112.3	146.1	141.1	143.1	140.7	152.5	133.5	145.0	123.9	123.5	118.7
**Fish**	23.8	31.3	25.5	35.7	19.2	29.3	22.9	17.6	17.7	27.3	26.7	23.4	17.0	17.2	18.1
**Dairy**	331.2	583.0	191.5	175.8	193.0	339.7	194.8	1292.1	163.5	188.5	406.4	379.5	245.3	125.2	184.8
**Oil**	1106.6	105.3	304.7	271.9	169.6	1552.7	459.3	324.1	260.1	424.0	1521.6	157.9	276.0	195.1	424.0
**Misc. Bev.**	4.8	4.7	4.4	4.8	6.1	4.4	4.4	4.6	4.6	5.9	4.0	4.4	4.2	4.4	6.0

Results are nationally representative with the use of survey weights.

**Table A14:** Vitamin B12 (mg) per USD

	Coastal					N. Highlands					Lake				
	2008	2010	2012	2014	2019	2008	2010	2012	2014	2019	2008	2010	2012	2014	2019
**Cereal**	0.1	0.1	0.1	0.1	0.1	0.1	0.1	0.1	0.1	0.1	0.2	0.1	0.1	0.1	0.1
**Starch**	0.0	0.0	0.0	0.0	0.0	0.0	0.0	0.0	0.0	0.0	0.0	0.0	0.0	0.0	0.0
**Sugar**	0.0	0.0	0.0	0.0	0.0	0.0	0.0	0.0	0.0	0.0	0.0	0.0	0.0	0.0	0.0
**Pulses/nuts**	0.0	0.0	0.0	0.0	0.0	0.0	0.0	0.0	0.0	0.0	0.0	0.0	0.0	0.0	0.0
**Veg**	0.0	0.0	0.0	0.0	0.0	0.0	0.0	0.0	0.0	0.0	0.0	0.0	0.0	0.0	0.0
**Fruit**	0.0	0.0	0.0	0.0	0.0	0.0	0.0	0.0	0.0	0.0	0.0	0.0	0.0	0.0	0.0
**Meat**	5.3	6.6	4.9	**5.8**	**9.6**	**7.5**	9.3	4.7	5.7	**9.7**	6.1	5.9	6.5	6.2	**10.0**
**Egg**	0.9	0.7	0.6	0.6	0.7	0.9	0.7	0.7	0.8	0.7	0.9	0.7	0.7	0.8	0.7
**Fish**	**8.5**	**8.8**	**6.9**	4.4	5.6	6.1	**9.4**	**7.4**	**7.1**	5.5	**12.2**	**9.5**	**7.3**	**8.0**	6.2
**Dairy**	3.0	2.2	2.2	1.6	1.7	2.9	2.1	2.1	2.3	1.9	3.8	2.7	2.5	2.6	2.3
**Oil**	0.1	0.0	0.0	0.0	0.0	0.0	0.0	0.0	0.0	0.1	0.1	0.0	0.0	0.0	0.1
**Misc. Bev.**	0.0	0.0	0.0	0.0	0.0	0.0	0.0	0.0	0.0	0.0	0.0	0.0	0.0	0.0	0.0
	**Central**					**S. Highlands**					**Southern**				
	**2008**	**2010**	**2012**	**2014**	**2019**	**2008**	**2010**	**2012**	**2014**	**2019**	**2008**	**2010**	**2012**	**2014**	**2019**
**Cereal**	0.1	0.2	0.1	0.2	0.1	0.1	0.1	0.1	0.1	0.1	0.1	0.1	0.2	0.1	0.1
**Starch**	0.0	0.0	0.0	0.0	0.0	0.0	0.0	0.0	0.0	0.0	0.0	0.0	0.0	0.0	0.0
**Sugar**	0.0	0.0	0.0	0.0	0.0	0.0	0.0	0.0	0.0	0.0	0.0	0.0	0.0	0.0	0.0
**Pulses/nuts**	0.0	0.0	0.0	0.0	0.0	0.0	0.0	0.0	0.0	0.0	0.0	0.0	0.0	0.0	0.0
**Veg**	0.0	0.0	0.0	0.0	0.0	0.0	0.0	0.0	0.0	0.0	0.0	0.0	0.0	0.0	0.0
**Fruit**	0.0	0.0	0.0	0.0	0.0	0.0	0.0	0.0	0.0	0.0	0.0	0.0	0.0	0.0	0.0
**Meat**	6.2	8.3	**8.4**	10.0	**9.9**	**11.1**	**18.9**	4.9	**10.8**	**10.0**	8.4	6.1	**6.5**	**5.7**	**9.5**
**Egg**	1.2	0.9	0.8	0.8	0.6	0.8	0.7	0.8	0.7	0.8	0.7	0.8	0.7	0.6	0.6
**Fish**	**7.6**	**10.0**	8.2	**11.4**	6.1	9.4	7.3	5.6	5.7	8.7	**8.6**	**7.5**	5.4	5.5	5.8
**Dairy**	4.1	5.8	2.4	2.2	2.3	3.6	2.5	**10.5**	2.0	2.2	3.9	3.8	2.7	1.5	2.2
**Oil**	0.1	0.0	0.0	0.0	0.0	0.2	0.1	0.0	0.0	0.1	0.2	0.0	0.0	0.0	0.1
**Misc. Bev.**	0.0	0.0	0.0	0.0	0.0	0.0	0.0	0.0	0.0	0.0	0.0	0.0	0.0	0.0	0.0

Results are nationally representative with the use of survey weights.

**Fig. A4:**
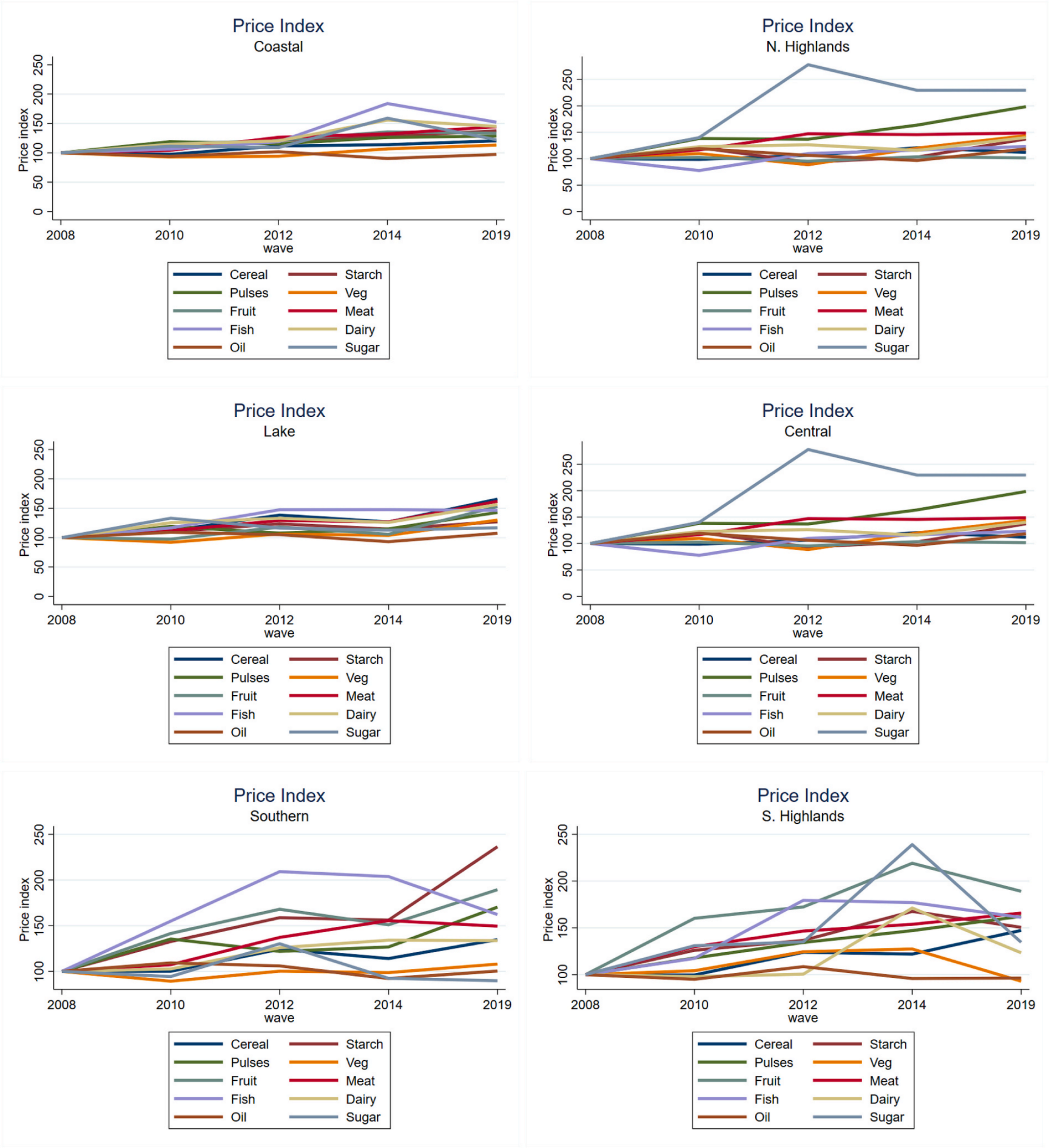
Food indexes by zone, 2008–2019..

**Table A.15:** Regression analysis, percentage change in adequacy index for energy, protein, and nutrients.

Adequacy index percentage change	Kcal	Protein	Calcium	Iron	Zinc	Vitamin A	Vitamin B12
Rural	−5.9***	−9.3***	−3.1***	−0.7	−6.2***	−13.4**	−17.9***
	(1.3)	(1.8)	(0.7)	(2.2)	(1.6)	(5.4)	(3.7)
HH members	−2.8***	−3.8***	−1.4***	−5.8***	−3.8***	−8.7***	−2.6***
	(0.1)	(0.2)	(0.1)	(0.3)	(0.2)	(0.6)	(0.3)
Cost per corresponding nutrient per USD							
Cereal	0.0*	0.2***	0	1.1***	1.2***	0.1	17
	(0.0)	(0.1)	(0.0)	(0.2)	(0.3)	(0.7)	(28.1)
Starches	−0.0*	−0.6***	0	−1.5***	−2.3***	0	0
	(0.0)	(0.2)	(0.0)	(0.4)	(0.7)	(0.0)	(.)
Pulses and nuts	0	−0.1	0	−0.6	−0.7***	0	0
	(0.0)	(0.1)	(0.0)	(0.5)	(0.2)	(0.0)	(.)
Veg	0.0***	0.4***	0.0***	1.8***	1.8***	0.0***	0
	(0.0)	(0.1)	(0.0)	(0.6)	(0.5)	(0.0)	(.)
Fruit	0	0.2	0.0*	6.9***	0	−0.4***	0
	(0.0)	(0.8)	(0.0)	(2.5)	(3.7)	(0.1)	(.)
Meat	0	0.1	0	6.4**	2.6***	0.4***	−1.8*
	(0.0)	(0.1)	(0.0)	(3.0)	(1.0)	(0.1)	(1.0)
Egg	0	−0.3	0	−6.4	0.4	−0.1	1.7
	(0.0)	(0.3)	(0.0)	(4.1)	(2.9)	(0.1)	(6.2)
Fish	0	0	−0.0***	−7.7***	−0.8*	−0.4	−0.1
	(0.0)	(0.1)	(0.0)	(1.4)	(0.4)	(0.3)	(0.5)
Dairy	0	−0.1	0	−4.9	−0.9**	0	−2
	(0.0)	(0.1)	(0.0)	(3.0)	(0.5)	(0.0)	(1.4)
Oil	−0.0**	−1	0	−98.4**	0	0	14.7
	(0.0)	(2.5)	(0.0)	(46.8)	(.)	(0.0)	(17.6)
Sugar	0	−2.1*	0	−2.7*	−7.2***	0	0
	(0.0)	(1.2)	(0.0)	(1.5)	(1.5)	(.)	(.)
Share of food budget away from home	−78.6***	−102.2***	−31.2***	−138.8***	−96.8***	−166.4***	−70.2***
	(2.7)	(3.8)	(1.6)	(5.1)	(3.3)	(8.8)	(5.7)
HH head non-farm labor (0/1)	11.9***	15.9***	6.3***	2.3	11.3***	36.9***	30.7***
	(1.1)	(1.8)	(0.7)	(2.0)	(1.4)	(5.1)	(4.0)
Constant	112.1***	131.9***	38.1***	187.1***	168.3***	75.3***	84.7***
	(6.4)	(8.5)	(3.4)	(12.2)	(11.8)	(26.6)	(7.4)

N = 16,626. All regressions are OLS. All models control for zone, month, and year. . ***p < 0.01, **p < 0.05, and *p < 0.10.

## Data Availability

The data is publically available from the World Bank

## Footnotes

1 These coefficients were calculated using a method from Jenkins (2019).
